# Palladium-catalyzed formation of oxazolidinones from biscarbamates: a mechanistic study

**DOI:** 10.3762/bjoc.7.33

**Published:** 2011-02-24

**Authors:** Benan Kilbas, Metin Balci

**Affiliations:** 1 Department of Chemistry, Middle East Technical University, 06531 Ankara, Turkey; 2 Department of Chemistry, Faculty of Sciences, Düzce University, 81620 Düzce, Turkey

**Keywords:** bicyclic endoperoxides, biscarbamates, oxazolidinone, Pd-catalyzed allylic reaction, singlet oxygen

## Abstract

Oxazolidinones can be synthesized starting from cyclic biscarbamates via a palladium-catalyzed reaction. To test the proposed mechanism of this reaction, first, bicyclonorcarene endoperoxides derived from cyano and carbomethoxy cycloheptatrienes were synthesized and converted into the corresponding diols. The reaction of diols with toluenesulfonyl isocyanate followed by a palladium catalyzed reaction furnished oxazolidinone derivatives in similar yields. It was shown that, if one face of the double bond is blocked by substituents such as H or CN, the reaction also takes place. On the basis of these results, it was assumed that an antiperiplanar orientation of the metal and nucleophile is not necessary to form oxazolidinones. The metal is probably bonded to the allylic system from the same face as the nucleophile.

## Introduction

**Scheme 1:**
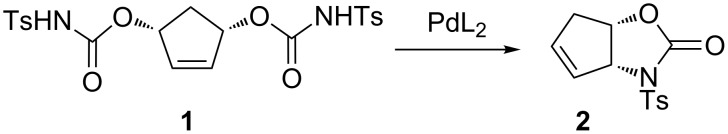
General route to oxazolidinones via a palladium-catalyzed reaction.

Palladium-catalyzed carbon–carbon bond formation reactions in synthetic organic chemistry have attracted considerable attention in recent years [1]. Palladium-catalyzed allylation is also a particularly useful method for the activation of allylic substrates [2]. Trost et al. described a highly stereoselective synthesis of oxazolidinone **2** starting from cyclic biscarbamate **1** via a palladium-catalyzed reaction, as shown in Scheme 1.

**Figure 1:**
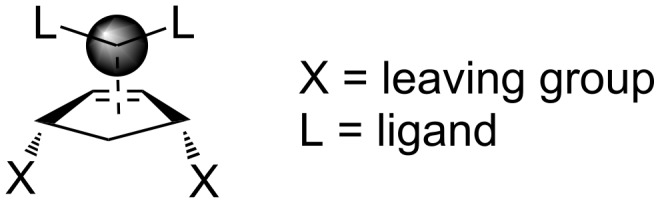
Metal–olefin complexation from the *anti*-face of the double bond.

The basic catalytic cyclization (Scheme 1) consists of metal–olefin complexation, ionization, substitution, and decomplexation. The tandem reaction sequence has been frequently applied in the synthesis of many complex rings and open chain systems containing diverse functionalities. It has been reported that the complexation takes place exclusively *anti* to the leaving group (Figure 1) [3–6].

**Figure 2:**
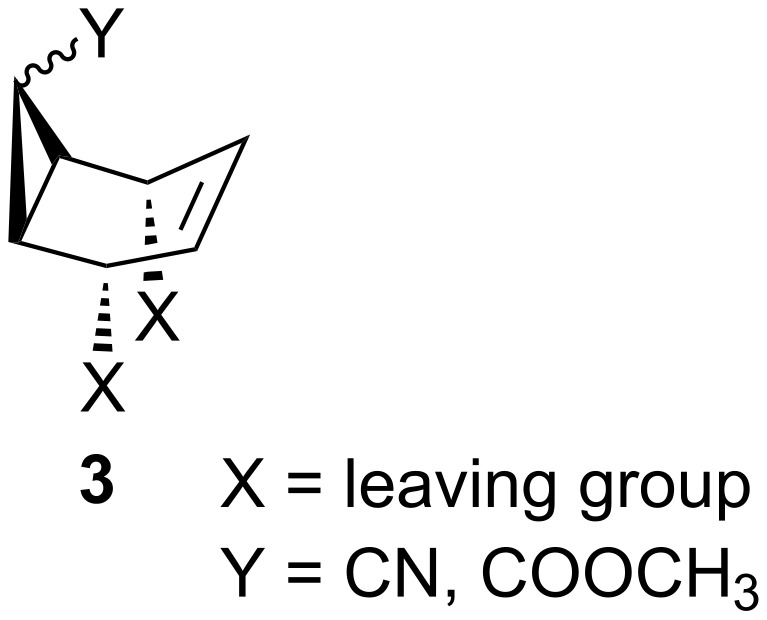
Cyclic systems with enantiotopic leaving groups at allylic positions.

On the other hand, Kurosawa et al. [7] and Greenberg et al. [8] reported that the palladium(0)–olefin and platinum(0)–olefin complexes add to 5-(methoxycarbonyl)-2-cyclohexenyl chloride and bromide from the *syn-*side of the leaving group and that the leaving group is capable of coordinating the metal to give the corresponding (η^3^-allyl)–palladium or –platinum complexes. In order to determine from which side the complexation takes place during oxazolidinone formation, we synthesized the cyclic systems **3** with enantiotopic leaving groups at allylic positions on different olefinic faces, where one face of the olefin can be blocked by the substituents Y (Figure 2).

## Results and Discussion

**Scheme 2:**
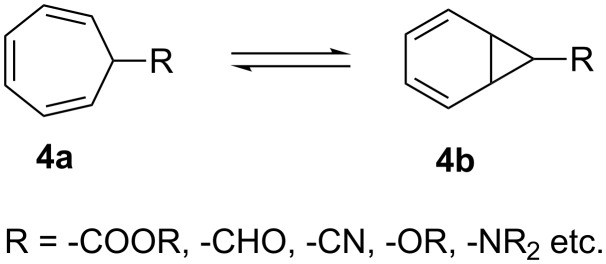
The cycloheptatriene–norcaradiene equilibrium.

The cycloheptatriene–norcaradiene (CHT–NOR) system **4a**
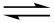
**4b** was an ideal starting point for construction of skeleton of **3**. The CHT–NOR **4a**
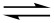
**4b** equilibrium has been substantially delineated by means of both physical and chemical methods (Scheme 2) [9–12].

**Scheme 3:**
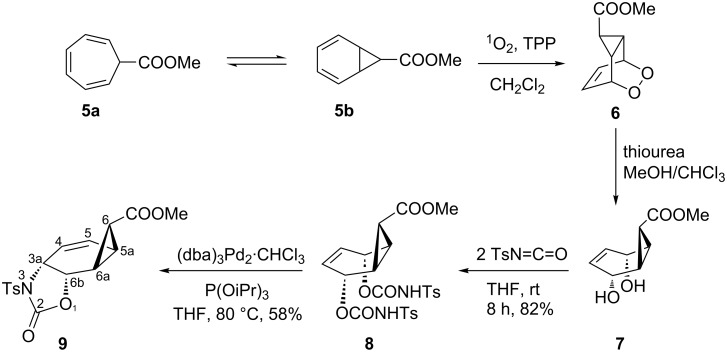
Synthesis of oxazolidinone **9** starting from carboxymethyl cycloheptatriene **5a**.

Electron accepting substituents, such as –CHO, –COOR, –CN etc. at C-7 tend to shift the equilibrium to the norcaradiene **4b** side, while electron donating substituents, such as –OR, –NR_2_ favor the cycloheptatriene **4a** structure. It has been shown that singlet oxygen and 4-phenyl-1,2,4-triazoline-3,5-dione (PTAD) are sufficiently reactive to intervene in the cycloheptatriene–norcaradiene equilibrium via cycloaddition [13–15]. For the construction of the skeleton **3**, compounds **5** and **11** were chosen as the starting materials (Scheme 3).

The ester **5** was obtained in high yield by the hydrolysis of cyanocycloheptatriene **11** as reported in the literature [16]. Tetraphenylporphyrin sensitized photooxygenation of the cycloheptatriene derivative **5a**
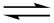
**5b** at room temperature resulted exclusively in the formation of the norcarene endoperoxide **6** [14,17]. The exact configuration of the endoperoxide **6** was determined by the single crystal X-ray analysis of the bisepoxide formed by the thermolysis of **6** [18]. Selective reduction of the peroxide linkage in **6** was carried out with thiourea under very mild conditions to give the diol **7** [19]. Since only the oxygen–oxygen bond is broken in this reaction, the configuration at all carbon atoms is preserved. Oxazolidinone **9** was synthesized by two consecutive reactions, i.e., the generation of **8** and a subsequent a stereospecific Pd(0)-catalyzed cyclization [3–5,20]. Thus, the ene-diol **7** was first treated with 2 equiv of toluenesulfonyl isocyanate to give the corresponding biscarbamate **8**. A solution of biscarbamate **8** was then added to a 5% solution of the palladium catalyst in THF, prepared from tris(dibenzylideneacetone)dipalladium chloroform complex and triisopropyl phosphite. The resulting oxazolidinone **9** was purified by chromatography on a silica gel column to give a crystalline product in 58% yield. The structure and configuration of **9** was assigned from ^1^H (COSY, HSQC, HMBC) and ^13^C NMR spectroscopic data. The most conspicuous features in the ^1^H NMR spectrum of this compound were the five-membered ring proton resonances. The proton H-6b adjacent to the oxygen atom resonates at 5.12 ppm as a doublet of doublets, (*J*_6b,3a_ = 7.8 Hz, *J*_6b,6a_ = 1.4 Hz). Geometry optimization calculations (MM2) on the molecule show a dihedral angle Φ of 71° for H-6a–H-6b in **9**, which is in agreement with the measured coupling constant *J*_6b,6a_ = 1.4 Hz. In case of a *syn*-configuration of the oxazolidinone ring one would expect a much larger coupling constant due to the calculated smaller dihedral angle (32°). This indicates the *anti-*configuration of the oxazolidinone ring in **9**. Furthermore, the observed large allylic coupling (*J*_3a,5_ = 1.7 Hz) also supports an *anti-*configuration. A maximum π-contribution to the allylic coupling is observed when Φ is 90° [21]. Our calculation for *anti-*configuration shows that the dihedral angle between the protons H-3a and H-5 is 67°, which is in agreement with the proposed configuration. In the case of *syn*-configuration, the calculated dihedral angle between H-3a and H-5 is 43°, which would give a smaller coupling constant. The measured coupling between the oxazolidinone ring protons (*J*_3a,6b_ = 7.8 Hz) shows the *cis*-relation between those protons.

**Scheme 4:**
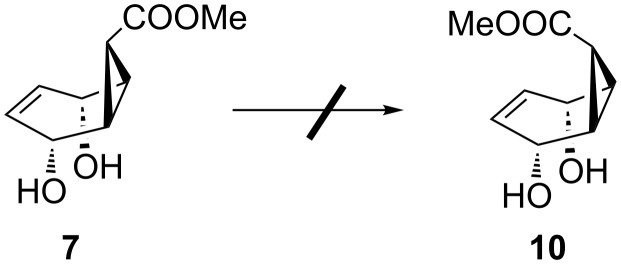
Attempted isomerization of **7** to **10**.

After the successful formation of the oxazolidinone derivative **9**, we tried to synthesize the *endo-*isomer **10**, where one face of the double bond is blocked by the bulky carboxylate group. Unfortunately, all efforts to isomerize the configuration of the carboxylate group in **7** failed (Scheme 4). After this attempted isomerization, we turned our attention to cyanocycloheptatriene (**11a**).

**Scheme 5:**
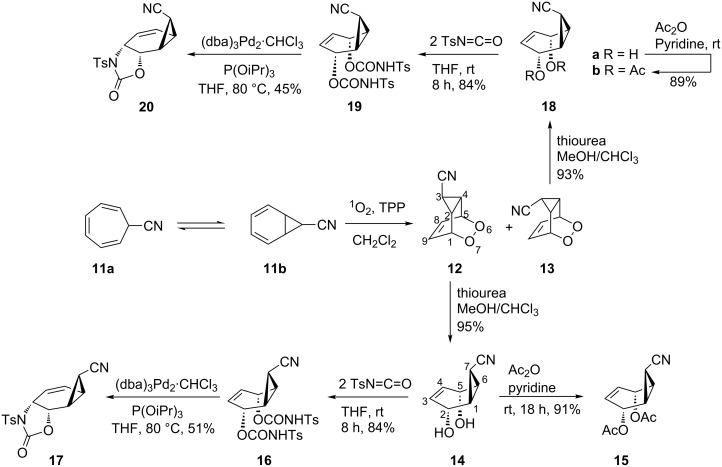
Synthesis of oxazolidinones **17** and **20**.

The starting material, 7-cyanocycloheptatriene **11a**, was synthesized by the reaction of the tropylium cation with cyanide anion as described in the literature [16]. The tetraphenylporphyrin sensitized photooxygenation of the cycloheptatriene derivative **11a**
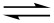
**11b** at room temperature gave a mixture of norcarene endoperoxides **12**–**13** in 42 and 33% yields, respectively (Scheme 5) [22]. The exact configuration of the cyano groups in **12** and **13** were determined by measuring the coupling constants between the cyclopropane protons. The cyclopropyl proton H-3 in **12** resonates as a triplet with a coupling constant of *J* = 3.3 Hz, whereas the *endo-*isomer **13** shows a coupling of 7.9 Hz. Since the *cis-*coupling in cyclopropane is larger than the *trans-*coupling [23–24], we assigned the *exo-*configuration to the cyano group in **12**.

Selective reduction of the peroxide linkages in **12** and **13** with thiourea under very mild conditions afforded the diols **14** and **18a**, respectively. For further characterization, the diols were converted to the diacetates **15** and **18b**. The isomeric diols **14** and **18a** were treated with 2 equiv of toluenesulfonyl isocyanate as described above to give the corresponding biscarbamates **16** and **19**. Treatment of **16** and **19** with the palladium catalyst (as described above) resulted in the formation of oxazolidinone derivatives **17** and **20** in 51 and 45% yield, respectively. Careful examination of the reaction mixture did not reveal the formation of any other isomers. The compounds were characterized by NMR spectroscopic data (COSY, HSQC, and HMBC). To determine the exact configuration of the oxazolidinone derivatives **17** and **20**, full assignments of all the protons were first made with the help of the COSY spectrum and the coupling constants determined. The coupling constants of the ring protons clearly support the fact that all three oxazolidinone derivatives **9**, **17** and **20** have the same configuration (Table 1).

**Table 1:** The coupling constants of compounds **9**, **17**, and **20**.

Compound	Coupling constants in Hz								
	*J*_4,5_	*J*_4,3a_	*J*_5,3a_	*J*_5,5a_	*J*_3a,6b_	*J*_6a,6b_	*J*_5a,6a_	*J*_6,5a_	*J*_6,6a_
	
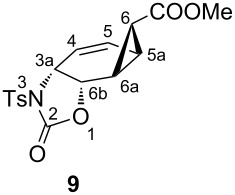	10.3	1.7	1.7	5.3	7.8	1.4	7.5	3.8	5.0
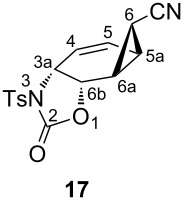	10.4	1.9	1.5	5.3	7.9	1.8	7.9	4.1	5.3
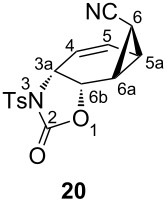	10.4	2.3	1.7	5.0	8.3	—^a^	—^a^	—^a^	—^a^

^a^These coupling constants could not be determined due to overlapping resonances.

According to the proposed mechanism of oxazolidinone formation [3], the first step is complex formation between the metal and olefin. Steric and electronic effects of the olefin determine the stability of the complex. For example, bulky groups decrease the stability of the complex via steric interactions, while electron-withdrawing groups will enhance the stability of the complex [25–26]. The next step is the ionization step followed by allylic substitution. The final step is the decomplexation.

**Scheme 6:**
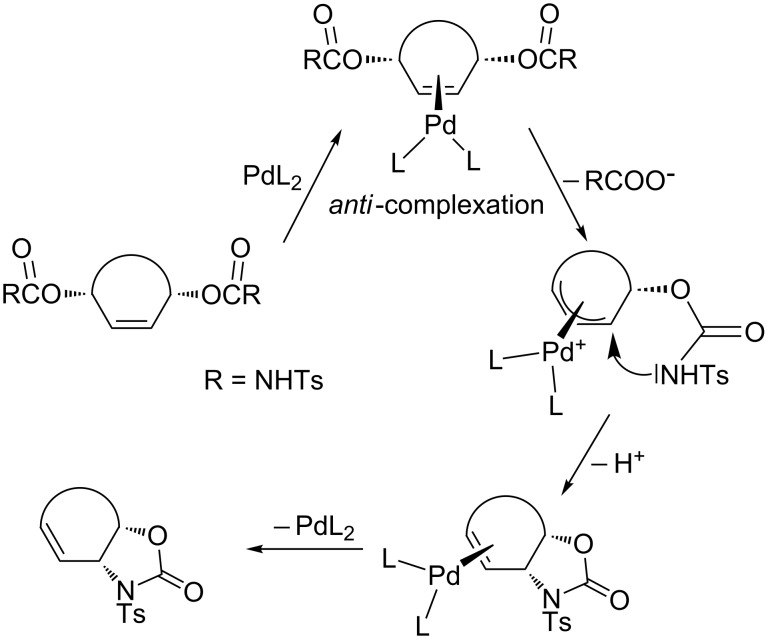
The mechanism for the formation of oxazolidinones [3].

Trost et al. [3] and Fiaud et al. [6] have proposed that only metal–olefin complexation *anti* to the leaving group will lead to the product (Scheme 6). In the case of **8**, **16** and **19**, the leaving groups, carbamates, are in *anti* (referred to the cyclopropane ring) positions and, therefore, the palladium complex should approach the double bond in **8**, **16**, and **19** from the side of the three-membered ring to form a complex.

**Figure 3:**
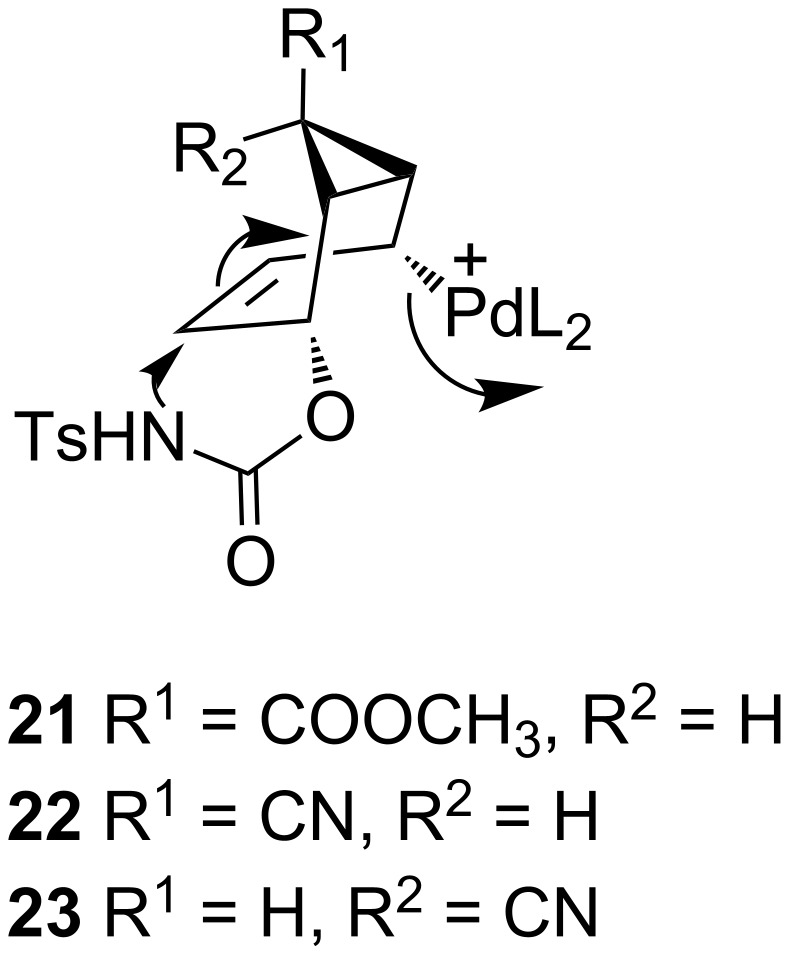
Nucleophilic attack on the double bond from the leaving group's side.

However, the cyclopropyl hydrogens H-6 in **8** and **16** are located over the six-membered ring. These hydrogen atoms block the *syn-*face of the double bond and may hinder the approach of the palladium complex from the side of the cyclopropane ring. However, the products **9** and **17** were formed under the same reaction conditions in 58 and 51% yield, respectively. If the proposed mechanism (Scheme 6) is correct, then the hydrogen atoms (H-6) in **8** and **16** cannot generate a steric hindrance to prevent the reaction. In order to increase the bulkiness of the substituents located over the six-membered ring, the hydrogen atom was replaced with the cyano group as in **19**. The reaction of **19** with the palladium complex was also unaffected or retarded; the product **20** was formed in a yield of 45%. In light of these results, we assume that complexation and ionization must be considered together. The removal of one of the enantiotopic leaving groups and complexation (from the side of the double bond) takes place at the same time. After the formation of the metal–carbon bond, the configuration of the metal is important. Fiaud et al. suggested an antiperiplanar orientation of metal and the nucleophile (–NHTs group) [6]. According to the S_N_2' substitution reaction mechanism [27–30], nucleophiles attack the double bond from the same side from which the leaving group departs, as shown below (Figure 3).

Therefore, we assume that metal is bonded to the allylic system from the same side as the nucleophile attacking the double bond to form the final product.

## Experimental

**General:** Melting points are uncorrected. Infrared spectra were obtained from KBr pellets on an FT-IR Bruker Vertex 70 instrument. The ^1^H and ^13^C NMR spectra were recorded on a Bruker BioSpin (DPX-400) instrument. Apparent splitting is given in all cases. Column chromatography was performed on silica gel (60-mesh, Merck), and TLC was carried out on Merck 0.2 mm silica gel 60 F_254_ analytical aluminum plates. Elemental analyses were carried out on a Leco-932 model CHNS analyzer.

***rel-*(1*R*,2S,5*S*,6*S*)-Methyl 2,5-bis(tosylcarbamoyloxy)bicyclo[4.1.0]hept-3-ene-7-carboxylate (8):** To a magnetically stirred solution of diol **7** [17] (1.00 g, 5.43 mmol) in THF (40 mL), was added a solution of *p*-toluenesulfonyl isocyanate (2.13 g, 10.88 mmol) in THF (5 mL) dropwise over 15 min at room temperature under a nitrogen atmosphere. The mixture was stirred at room temperature for 8 h. The solvent was then removed under reduced pressure. The residue was purified by rapid filtration through silica gel (110 g) with hexane/ethyl acetate (1:1) as eluent. Evaporation of solvent gave white crystals (2.57 g, 82%, mp 176–178 °C). ^1^H NMR (400 MHz, CDCl_3_) δ 8.39 (br s, –NH, 2H), 7.91 (d, *J* = 8.0 Hz, 4H, aromatic)), 7.32 (d, *J* = 8.0 Hz, 4H, aromatic), 5.92 (br s, 2H, H-3 and H-4), 5.39–5.40 (m, 2H, H-2 and H-5), 3.66 (s, 3H, –OCH_3_), 2.44 (s, 6H, –CH_3_), 1.94 (d, *J* = 4.0 Hz, 2H, H-1 and H-6), 1.43 (t, *J* = 4.0 Hz, H-7); ^13^C NMR (100 MHz, CDCl_3_) δ 171.6 (C=O), 150.1 (C=O), 145.2, 135.4, 129.7, 128.5, 127.5, 66.2 (C-2 and C-5), 52.4 (–OCH_3_), 21.7, 21.6, 21.0; IR (ν_max_, cm^−1^) 3209, 1749, 1673, 1447, 1349, 1317, 1203, 1183, 1088, 862, 838, 662. Anal. Calcd for C_25_H_26_N_2_O_10_S_2_: C, 51.89; H, 4.53; N, 4.84; S, 11.08. Found: C, 52.13; H, 4.65; N, 4.91; S, 11.26.

***rel-*(3a*R,*5a*R,*6*R*,6a*S,*6b*S*)-Methyl 2-oxo-3-tosyl-3,3a,5a,6,6a,6b-hexahydro-2*H*-cyclopropa[3,4]benzo[1,2-*d*][1,3]oxazole-6-carboxylate (9):** Tris(dibenzylideneacetone)dipalladium chloroform complex (0.2 g, 193.6 mmol) was dissolved in freshly distilled THF (20 mL) under a nitrogen atmosphere. Triisopropyl phosphite (1.6 mL) in THF (5 mL) was then added. The mixture was stirred at room temperature until a clear green solution was obtained. The prepared catalyst solution was added to a stirred solution of the biscarbamate **8** (1.00 g, 1.73 mmol) in THF (50 mL). The reaction was heated under reflux at 80 °C for 1 day. After completion of the reaction, the solvent was removed under reduced pressure. The residue was purified by rapid filtration through silica gel (100 g) with hexane/ethyl acetate (3:2) as eluent. After evaporation of solvent, the product was recrystallized from ethyl acetate/hexane as colorless crystals (365 mg, 58%, mp 167–169 °C). ^1^H NMR (400 MHz, CDCl_3_) δ 7.95 (d, *J* = 8.1 Hz, 2H), 7.35 (d, *J* = 8.1 Hz, 2H), 6.26 (ddd, *J*_5,4_ = 10.3, *J*_5,5a_ = 5.3, *J*_5,3a_ = 1.8 Hz, A-part of AB system, H-5), 5.62 (dd, *J*_4,5_ = 10.3, *J*_4,3a_ = 1.7 Hz, B-part of AB system, H-4), 5.12 (dd, *J*_6b,3a_ = 7.8, *J*_6b,6a_ = 1.4 Hz, A-part of AB system, H-6b), 4.70 (dt, *J*_3a,6b_ = 7.8, *J*_3a,4_ = *J*_3a,5_ = 1.7 Hz, B-part of AB system, H-3a), 3.70 (s, 3H, –OCH_3_), 2.45 (s, 3H, –CH_3_), 2.27 (ddd, *J*_6a,5a_ = 7.5, *J*_6a,6_ = 5.0, *J*_6a,6b_ = 1.4 Hz, H-6a), 2.07 (ddd, *J*_5a,6a_ = 7.5, *J*_5a,5_ = 5.3, *J*_5a,6_ = 3.8 Hz, H-5a), 1.57 (dd, *J*_6,6a_ = 5.0 *J*_6,5a_ = 3.8 Hz, H-6); ^13^C NMR (100 MHz, CDCl_3_) δ 171.0 (C=O), 150.9 (C=O), 145.9, 135.6, 130.1, 129.2 (C-5), 128.7, 119.9 (C-4), 70.5 (C-6b), 53.2 (C-3a), 52.6 (OCH_3_), 29.7 (C-6), 21.9 (Ar-CH_3_), 20.5 (C-6a), 18.9 (C-5a); IR (*ν*_max_, cm^−1^) 1770, 1715, 1365, 1290, 1164, 1080, 1067, 1049, 671. Anal. Calcd for C_17_H_17_NO_6_S: C, 56.19; H, 4.72; N, 3.85; S, 8.82. Found: C, 56.08; H, 4.67; N, 3.85; S, 8.63.

***rel*-(1*R*,2*S*,5*R*,6*S*)-2,5-Dihydroxybicyclo[4.1.0]hept-3-ene-7-*exo-*carbonitrile (14):** To a magnetically stirred solution of endoperoxide **12** (1.00 g, 6.71 mmol) in MeOH/CHCl_3_ solution (30 mL), was added thiourea (513 mg, 6.75 mmol) over a 10 min period at room temperature and the mixture stirred at the same temperature for 2 h. The residue was filtered through filter paper. Evaporation of solvent under reduced vacuum gave diol **14** as colorless crystals (0.96 g, 95%, mp 139–141 °C). ^1^H NMR (400 MHz, DMSO-*d*_6_) δ 5.60 (quasi d, *J* = 2.4 Hz, 2H, H-3 and H-4), 4.98 (d, *J* = 8.0 Hz, 2H, –OH), 4.17 (br d, *J* = 7.6, 2H, H-2 and H-5), 1.85 (d, *J* = 4.8 Hz, 2H, H-1 and H-6), 1.23 (t, *J* = 4.8 Hz, H-7); ^13^C NMR (100 MHz, DMSO-*d*_6_) δ 127.0 (C-3 and C-4), 121.3 (CN), 59.8 (C-2 and C-5), 25.0 (C-1 and C-6), 2.5 (C-7); IR (*ν*_max_, cm^−1^) 3257, 2927, 2234, 1481, 1306, 1094, 1015, 777. Anal. Calcd for C_8_H_9_NO_2_: C, 63.56; H, 6.00; N, 9.27. Found: C, 63.38; H, 5.87; N, 9.42.

***rel-*(1*R*,2*S*,5*R*,6*S*)-5-(Acetyloxy)-7-*exo-*cyanobicyclo[4.1.0]hept-3-en-2-yl acetate** (**15**): A mixture of diol **14** (0.96 g, 6.35 mmol), acetic anhydride (4 mL) and pyridine (6 mL) was stirred at room temperature for 18 h. The mixture was then cooled to 0 °C, diluted with water, neutralized with aqueous HCl and extracted with ethyl acetate. The combined organic layer was washed with saturated NaCl, dried over Na_2_SO_4_ and concentrated under reduced pressure. Recrystallization of residue from ethyl acetate/hexane gave colorless crystals (1.36 g, 91%, mp 103–105 °C). ^1^H NMR (400 MHz, CDCl_3_) δ 5.89–5.87 (m, 2H, H-3 and H-4), 5.40–5.43 (m, 2 H, H-2 and H-5), 2.05 (s, 6H, –CH_3_), 2.03 (d, *J* = 4.8 Hz, 2H, H-1 and H-6), 1.16 (t, *J* = 4.8 Hz, H-7); ^13^C NMR (100 MHz, CDCl_3_) δ 170.3 (C=O), 126.3 (C-3 and C-4), 119.2 (–CN), 62.8 (C-2 and C-5), 21.5 (C-1 and C-6), 21.0 (–CH_3_), 3.6 (C-7); IR (*ν*_max_, cm^−1^) 2238, 1727, 1369, 1226, 1052, 1015, 990, 968. Anal. Calcd for C_12_H_13_NO_4_: C, 61.27; H, 5.57; N, 5.95. Found: C, 61.20; H, 5.44; N, 6.04.

***rel*-(1*R*,2*S*,5*R*,6*S*)-2,5-Dihydroxybicyclo[4.1.0]hept-3-ene-7-*endo-*carbonitrile (18a)**: A solution of endoperoxide **13** (1.00 g, 6.71 mmol) and thiourea (513 mg, 6.75 mmol) in MeOH/CHCl_3_ (30 mL) was reacted as described above. After evaporation of solvent under reduced pressure, diol **18a** was obtained as colorless crystals (0.94 g, 93%, mp 160–162 °C). ^1^H NMR (400 MHz, methanol-*d*_4_) δ 5.73 (br s, 2H, H-3 and H-4), 4.7 (br s, –OH) 4.17 (br s, 2H, H-2 and H-5), 1.84–1.71 (m, AB_2_ system, 3H, cyclopropane); ^13^C NMR (100 MHz, methanol-*d*_4_) δ 129.5 (C-3 and C-4), 118.9 (–CN), 61.4 (C-2 and C-5), 23.0 (C-1 and C-6), 4.1 (C-7); IR (*ν*_max_, cm^−1^) 3236, 3050, 2238, 1022, 993, 980. Anal. Calcd for C_8_H_9_NO_2_: C, 63.56; H, 6.00; N, 9.27. Found: C, 63.47; H, 5.77; N, 9.33.

***rel-*(1*R*,2*S*,5*R*,6*S*)-5-(Acetyloxy)-7-*endo-*cyanobicyclo[4.1.0]hept-3-en-2-yl acetate (18b)**: A mixture of diol **18a** (0.94 g, 6.22 mmol), acetic anhydride (4 mL) and pyridine (6 mL) was stirred at room temperature for 16 h. The mixture was then cooled to 0 °C, diluted with water, neutralized with aqueous HCl and extracted with ethyl acetate. The combined organic layer was washed with saturated NaCl, dried over Na_2_SO_4_ and concentrated under reduced pressure. Recrystallization of residue from ethyl acetate/hexane gave colorless crystals (1.30 g, 89%, mp 119–121 °C).^1^H NMR (400 MHz, CDCl_3_) δ 6.00 (br s, 2H, H-3 and H-4 ), 5.49 (br s, 2H, H-2 and H-5_5_), 2.11 (s, 6H, –CH_3_), 1.83 (s, 3H, cyclopropane); ^13^C NMR (100 MHz, CDCl_3_) δ 170.2 (C=O), 126.9 (C-3 and C-4), 116.3 (-CN), 62.2 (C-2 and C-5), 21.1 (–CH_3_), 18.9 (C-1 and C-6), 3.7 (C-7); IR (*ν*_max_, cm^−1^) 2225, 1722, 1367, 1230, 1034, 986, 977. Anal. Calcd for C_12_H_13_NO_4_: C, 61.27; H, 5.57; N, 5.95. Found: C, 61.22; H, 5.40; N, 5.96.

***rel-*(1*R*,2*R*,5*S*,6*S*)-7-*exo*-Cyanobicyclo[4.1.0]hept-3-ene-2,5-diyl bis(tosylcarbamate) (16):** A solution of diol **14** (0.96 g, 6.35 mmol) and *p*-toluenesulfonyl isocyanate (2.48 g, 12.71 mmol) in THF (40 mL) was reacted as described above. The product was obtained as white crystals (2.85 g, 84%, 155–157 °C). ^1^H NMR (400 MHz, DMSO-*d*_6_) δ 7.66 (d, *J* = 8.0 Hz, 4H, aromatic), 7.23 (d, *J* = 8.0 Hz, 4H, aromatic), 5.64 (br s, 2H, H-3 and H-4), 5.02 (br s, 2H, H-2 and H-5), 2.33 (s, 6H), 1.82 (br d, *J* = 4.8 Hz, 2H, H-1 and H-6), 1.40 (t, *J* = 4.8 Hz, H-7); ^13^C NMR (100 MHz, DMSO-*d*_6_) δ 154.5 (C=O), 139.6, 138.0, 126.2, 124.5, 123.6, 118.5 (–CN), 59.9 (C-2 and C-5), 19.6, 18.6, 0.2; IR (*ν*_max_, cm^−1^) 3260, 2240, 1745, 1435, 1349, 1208, 1184, 1160, 1089, 861, 829, 665. Anal. Calcd for C_24_H_23_N_3_O_8_S_2_: C, 52.83; H, 4.25; N, 7.70; S, 11.75. Found: C, 53.17; H, 4.51; N, 7.86; S, 11.98.

***rel-*(1*R*,2*R*,5*S*,6*S*)-7-*endo-*Cyanobicyclo[4.1.0]hept-3-ene-2,5-diyl bis(tosylcarbamate) (19):** A solution of diol **18a** (0.94 g, 6.22 mmol) and *p*-toluenesulfonyl isocyanate (2.43 g, 12.44 mmol) in THF (40 mL) was reacted as described above. The product was obtained as white crystals (2.75 g, 81%, 164–166 °C). ^1^H NMR (400 MHz, acetone-*d*_6_) 7.78 (d, *J* = 8.0 Hz, 4H, aromatic), 7.31 (d, *J* = 8.0 Hz, 4H, aromatic), 5.80 (br s, 2H, H-3 and H-4), 5.15 (m, 2H, H-2 and H-5), 2.31 (s, 6H), 1.99 (t, *J* = 9.2 Hz, H-7), 1.72 (d, *J* = 9.2 Hz, 2H, H-1 and H-6); ^13^C NMR (100 MHz, acetone-*d*_6_) δ 151.5 (C=O), 145.6, 137.7, 130.4, 129.0, 127.7, 126.9 (–CN), 65.3 (C-2 and C-5), 21.5, 19.2, 4.1 (C-7); IR (*ν*_max_, cm^−1^) 3355, 3259, 2231, 1742, 1439, 1350, 1220, 1158, 1086, 665. Anal. Calcd for C_24_H_23_N_3_O_8_S_2_: C, 52.83; H, 4.25; N, 7.70; S, 11.75. Found: C, 53.21; H, 4.46; N, 7.79; S, 11.91.

***rel-*3a*R,*5a*R,*6*R*,6a*S,*6b*S*-2-Oxo-3-tosyl-3,3a,5a,6,6a,6b-hexahydro-2*H*-cyclopropa[3,4]benzo[1,2-*d*][1,3]oxazole-7-*exo-*carbonitril (17):** Biscarbamate **16** (1.00 g, 1.83 mmol) was reacted with freshly prepared Pd-complex as described above. The product was recrystallized from ethyl acetate/hexane to afford colorless crystals (308 mg, 51%, mp 145–147 °C). ^1^H NMR (400 MHz, CDCl_3_) δ 7.94 (d, *J* = 8.3 Hz, 2H, aromatic), 7.44 (d, *J* = 8.3 Hz, 2H, aromatic), 6.28 (ddd, *J*_5,4_ = 10.4, *J*_5,5a_ = 5.3, *J*_5,3a_ = 1.5 Hz, H-5), 5.73 (dd, *J*_4,5_ = 10.4, *J*_4,3a_ = 1.9 Hz, H-4), 5.15 (br d, *J*_6b,3a_ = 7.9 Hz, H-6b), 4.67 (dt, *J*_3a,6b_ = 7.9, *J*_3a,5_ = *J*_3a,4_ =1.9 Hz, H-3a), 2.46 (s, 3H, –CH_3_), 2.30 (ddd, *J*_6a,5a_ = 7.9, *J*_6a,6_ = 5.3, *J*_6a,6b_ = 1.8 Hz, H-6a), 2.20 (dt, *J*_5a,6a_ = 7.9 Hz, *J*_5a,6_ = *J*_5a,5_ = 5.3 Hz, H-5a), 1.26 (dd, *J*_6,5a_ = 4.1, *J*_6,6a_ = 5.3 Hz, H-6); ^13^C NMR (100 MHz, CDCl_3_) *δ* 150.4 (C=O), 143.3 (C-aromatic), 135.3 (C-aromatic), 130.2 (CH-aromatic), 128.7 (CH-arom), 127.6 (C-5), 121.3 (C-4), 118.1 (–CN), 69.2 (C-6b), 52.5 (C-3a), 22.0 (–CH_3_), 19.9 (C-6b), 17.9 (C-5a), 12.7 (C-6); IR (*ν*_max_, cm^−1^) 2241, 1767, 1343, 1331, 1186, 1164, 1116, 1091, 841, 668. Anal. Calcd for C_16_H_14_N_2_O_4_S: C, 58.17; H, 4.27; N, 8.48; S, 9.71. Found: C, 58.13; H, 4.31; N, 8.41; S, 9.3.

***rel-*3a*R,*5a*R,*6*R*,6a*S,*6b*S*-2-Oxo-3-tosyl-3,3a,5a,6,6a,6b-hexahydro-2*H*-cyclopropa[3,4]benzo[1,2-*d*][1,3]oxazole-7-*endo-*carbonitril (20):** Biscarbamate **16** (1.00 g, 1.83 mmol) was reacted with freshly prepared Pd-complex as described above. The product was recrystallized from ethyl acetate/hexane to give colorless crystals (272 mg, 45%, mp 159–161 °C). ^1^H NMR (400 MHz, CDCl_3_) δ 7.96 (d, *J* = 8.3 Hz, 2H, aromatic), 7.37 (d, *J* = 8.3 Hz, 2H, aromatic), 6.19 (br dd, *J*_5,4_ = 10.4, *J*_5,5a_ = 5.0 Hz, H-5), 6.08 (dd, *J*_4,5_ = 10.4, *J*_4,3a_ = 2.3 Hz, H-4), 5.08 (br d, *J*_6b,3a_ = 8.3 Hz, H-6b), 4.86 (ddd, *J*_3a,6b_ = 8.3, *J*_3a,4_ = 2.3, *J*_3a,5_ = 1.7 Hz, H-3a), 2.46 (s, 3H, –CH_3_), 2.18–2.04 (m, AB_2_ system, 3H, cyclopropane); ^13^C NMR (100 MHz, CDCl_3_) δ 150.2 (C=O), 145.9 (C-aromatic), 135.1 (C-aromatic), 129.9 (CH-aromatic), 128.4 (CH-aromatic), 124.2 (C-5), 123.3 (C-4), 116.5 (CN), 68.7 (C-6b), 52.9 (C-3a), 21.7 (–CH_3_, 18.2 (cyclopropane), 15.6, (cyclopropane) 11.8 (cyclopropane); IR (*ν*_max_, cm^−1^) 2208, 1779, 1350, 1329, 1170, 1160, 1121, 1091, 1031, 667. Anal. Calcd for C_16_H_14_N_2_O_4_S: C, 58.17; H, 4.27; N, 8.48; S, 9.71. Found: C, 57.92; H, 4.29; N, 8.39; S, 9.47.

## Supporting Information

Supporting Information contains the ^1^H and ^13^C NMR spectra of all newly synthesized compounds. In three cases, COSY, DEPT-90^o^, DEPT-135^o^, HSQC and HMBC spectra are also given.

File 1 Supplementary data.
