# Supplementary material for: Palladium-catalyzed formation of oxazolidinones from biscarbamates: a mechanistic study
Source: Beilstein J Org Chem. 2011 Feb 24;7:246–53. doi: 10.3762/bjoc.7.33 (PMC3063050; doi:10.3762/bjoc.7.33)
Supplement: File 1 — Supplementary data. [file Beilstein_J_Org_Chem-07-246-s001.pdf]

Supporting Information

for

Palladium-catalyzed formation of oxazolidinones from biscarbamates: a mechanistic study

Benan Kilbas[1,2] and Metin Balci*[1]

Address: [1]Department of Chemistry, Middle East Technical University, 06531 Ankara, Turkey and [2]Department of Chemistry, Faculty of Sciences, Düzce University, 81620 Düzce, Turkey

Email: Metin Balci - mbalci@metu.edu.tr; Benan Kilbas - bkilbas@gmail.com

*Corresponding author

Supplementary data

Table of Contents

Title	Page
^{1}H NMR spectrum for **8**	3
^{13}C NMR spectrum for **8**	3
^{1}H NMR spectrum for **9**	4
^{13}C NMR spectrum for **9**	4
DEPT-90 spectrum for **9**	5
DEPT-135 spectrum for **9**	5
COSY spectrum for **9**	6
HSQC spectrum for **9**	6
HMBC spectrum for **9**	7
^{1}H NMR spectrum for **14**	8
^{13}C NMR spectrum for **14**	8

^{1}H NMR spectrum for **15** 9
^{13}C NMR spectrum for **15** 9
^{1}H NMR spectrum for **16** 10
^{13}C NMR spectrum for **16** 10
^{1}H NMR spectrum for **17** 11
^{13}C NMR spectrum for **17** 11
DEPT-90 spectrum for **17** 12
DEPT-135 spectrum for **17** 12
COSY spectrum for **17** 13
HSQC spectrum for **17** 13
HMBC spectrum for **17** 14
^{1}H NMR spectrum for **18a** 15
^{13}C NMR spectrum for **18a** 15
^{1}H NMR spectrum for **18b** 16
^{13}C NMR spectrum for **18b** 16
^{1}H NMR spectrum for **19** 17
^{13}C NMR spectrum for **19** 17
^{1}H NMR spectrum for **20** 18
^{13}C NMR spectrum for **20** 18
DEPT-90 spectrum for **20** 19
DEPT-135 spectrum for **20** 19
COSY spectrum for **20** 20
HSQC spectrum for **20** 20
HMBC spectrum for **20** 21

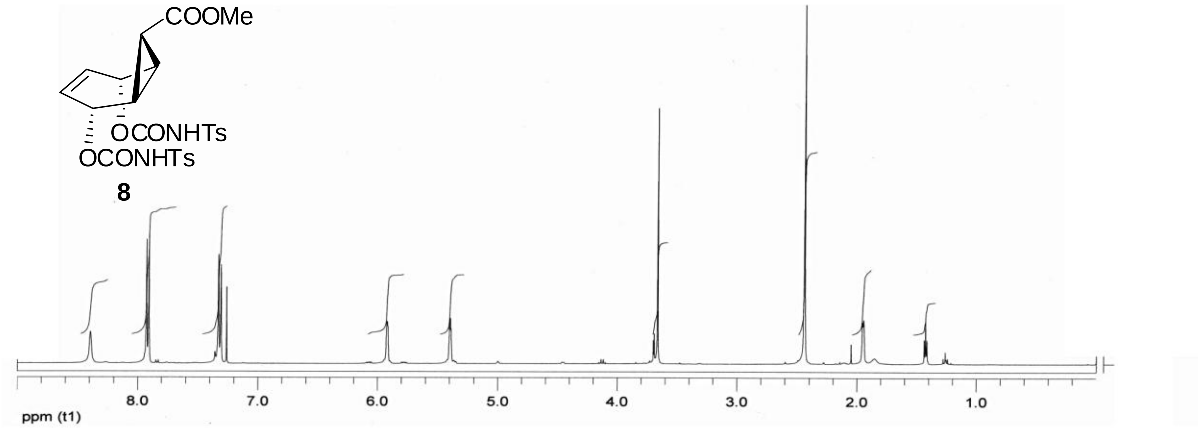
COOMe
OCONHTs
OCONHTs
8
ppm (t1)
8.0
7.0
6.0
5.0
4.0
3.0
2.0
1.0

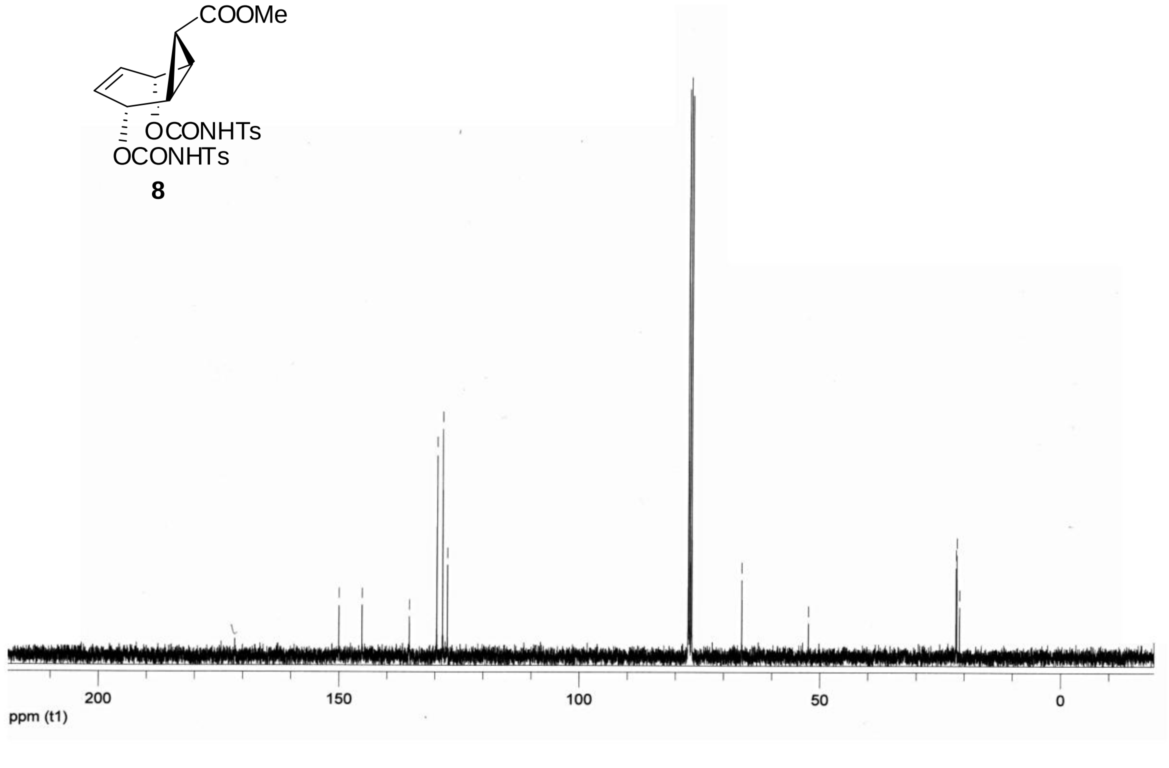
COOMe
OCONHTs
OCONHTs
8
ppm (t1)
200
150
100
50
0

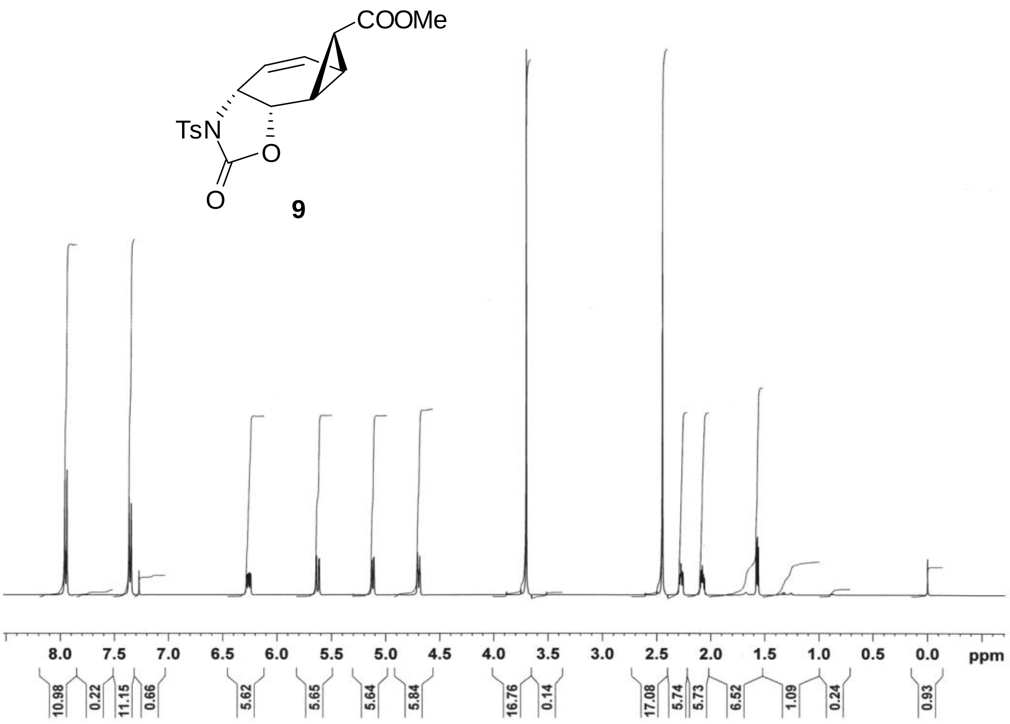
COOMe
TsN
O
O
9
8.0 7.5 7.0 6.5 6.0 5.5 5.0 4.5 4.0 3.5 3.0 2.5 2.0 1.5 1.0 0.5 0.0 ppm
10.98 0.22 11.15 0.66 5.62 5.65 5.64 5.84 16.76 0.14 17.08 5.74 5.73 6.52 1.09 0.24 0.93

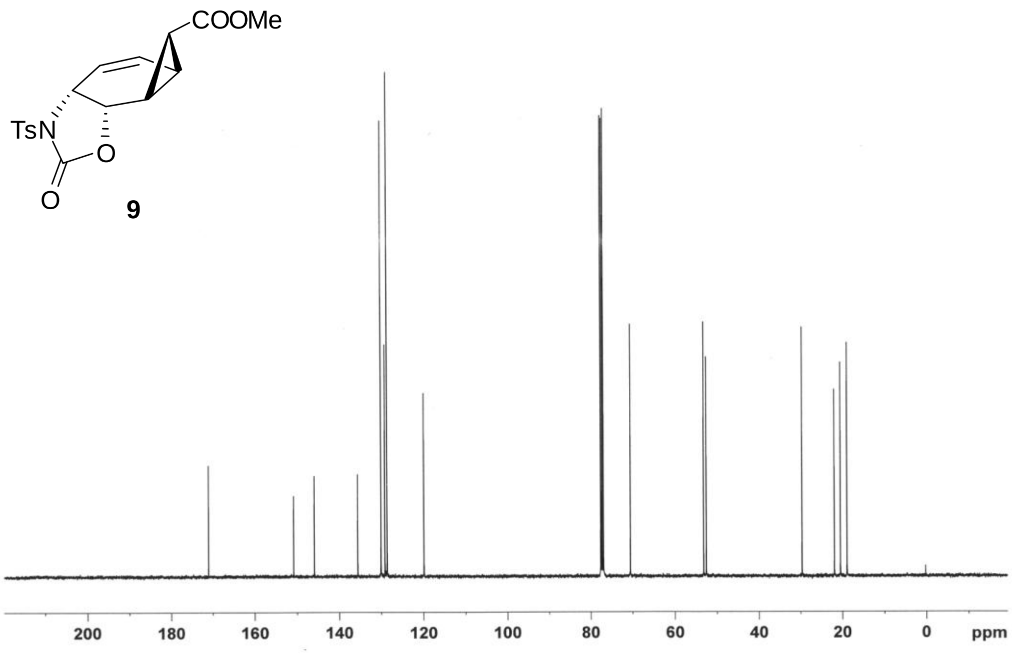
COOMe
TsN
O
O
9
200 180 160 140 120 100 80 60 40 20 0 ppm

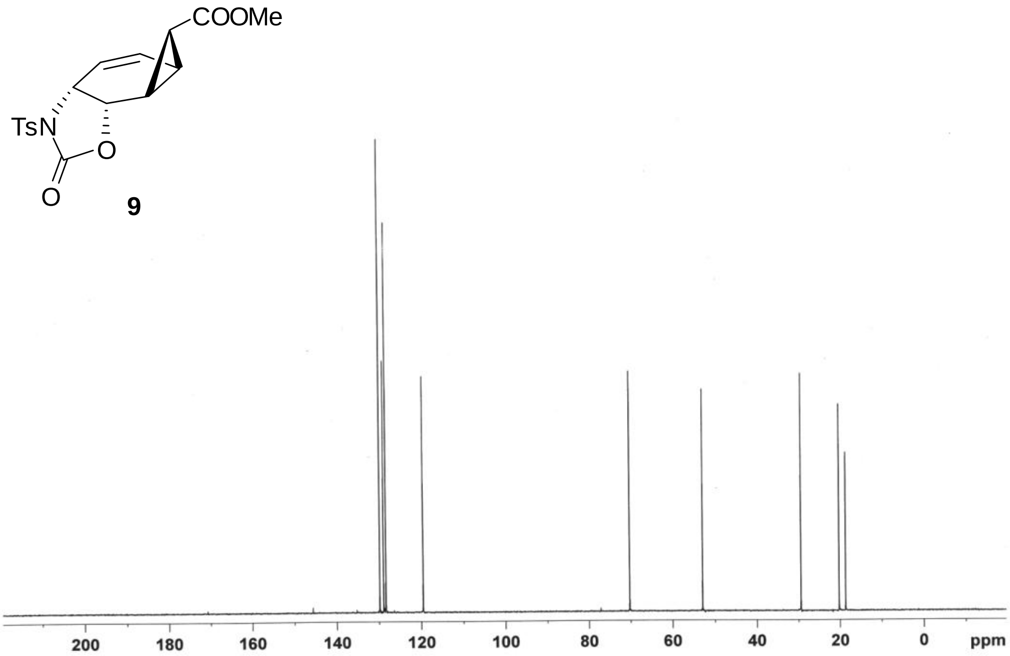
COOMe
TsN
O
O
9
200
180
160
140
120
100
80
60
40
20
0
ppm

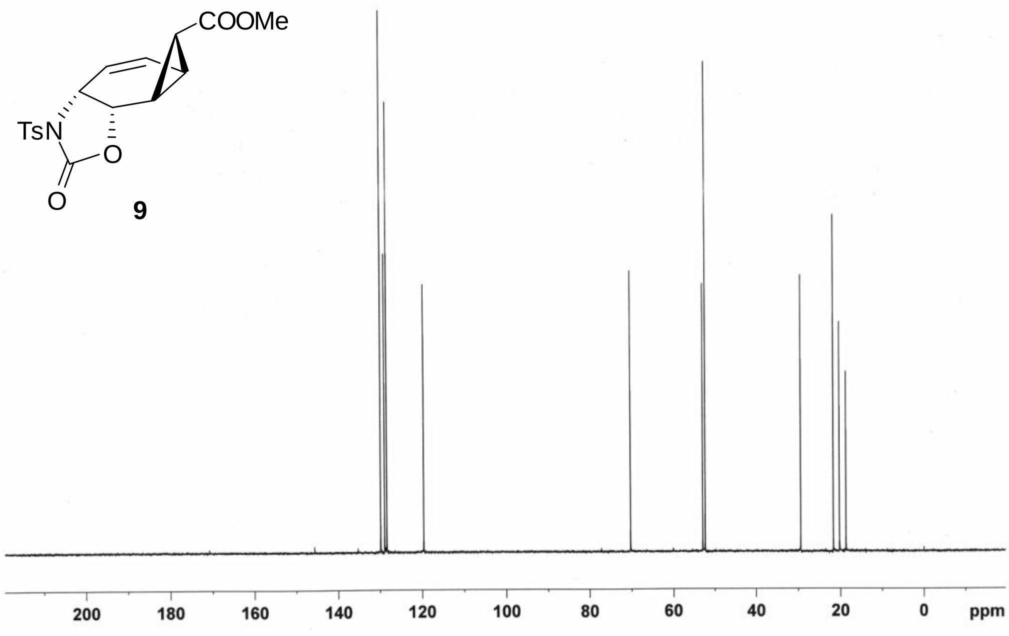
COOMe
TsN
O
O
9
200
180
160
140
120
100
80
60
40
20
0
ppm

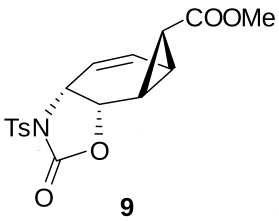
COOMe
TsN
O
O

9

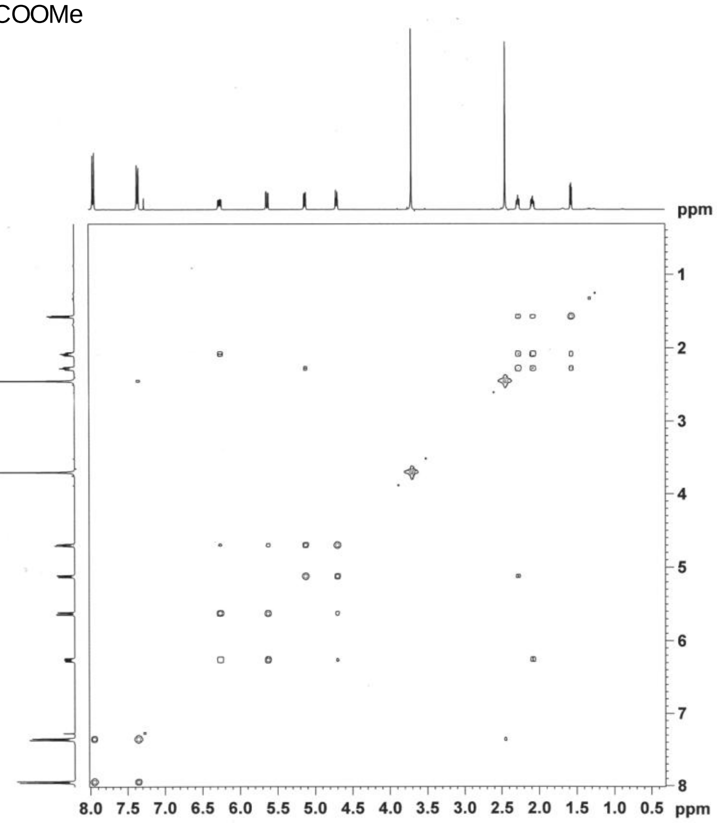
ppm
1
2
3
4
5
6
7
8
8.0 7.5 7.0 6.5 6.0 5.5 5.0 4.5 4.0 3.5 3.0 2.5 2.0 1.5 1.0 0.5 ppm

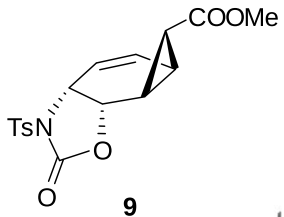
COOMe
TsN
O
O

9

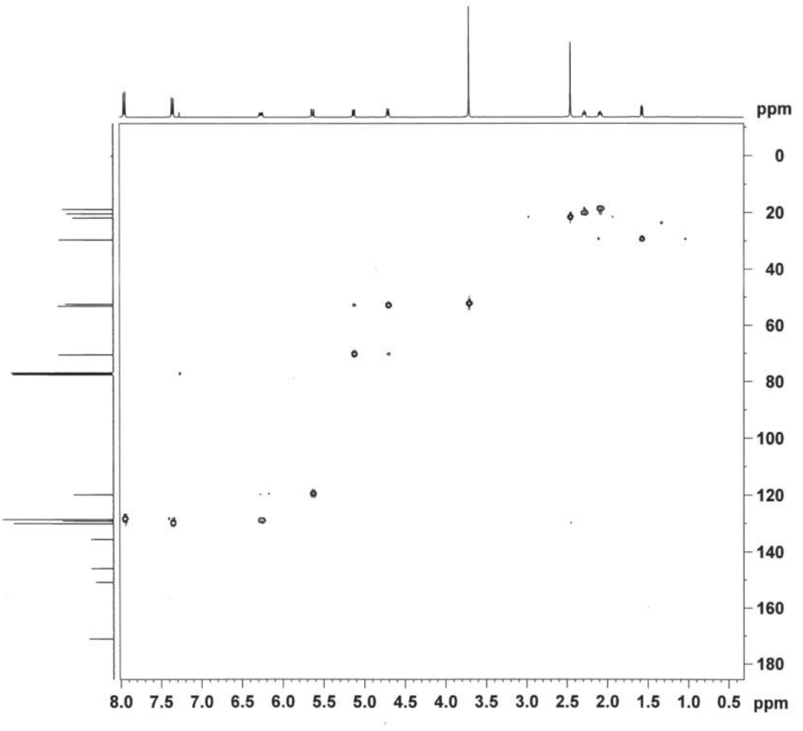
ppm
0
20
40
60
80
100
120
140
160
180
8.0 7.5 7.0 6.5 6.0 5.5 5.0 4.5 4.0 3.5 3.0 2.5 2.0 1.5 1.0 0.5 ppm

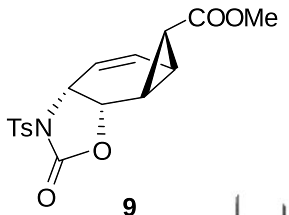
COOMe
TsN
O
O

9

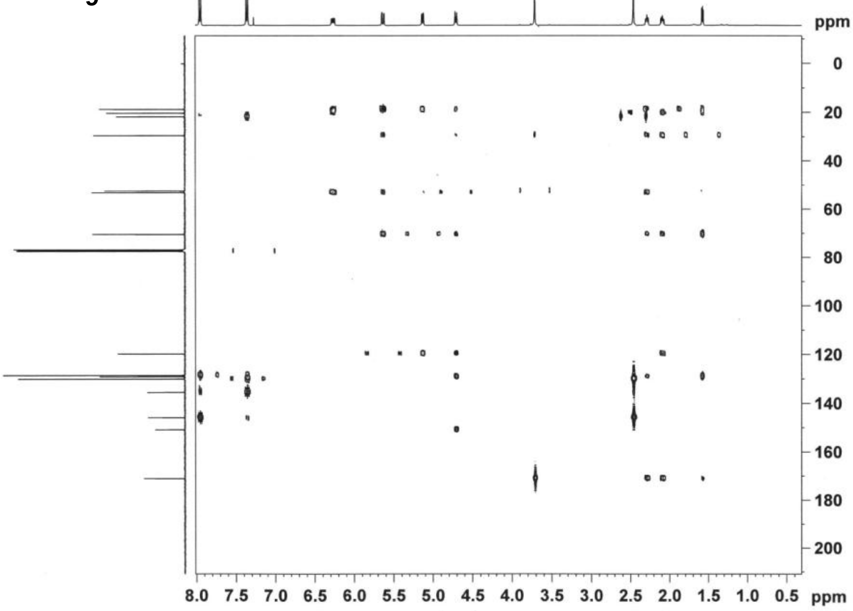
ppm
0
20
40
60
80
100
120
140
160
180
200
8.0 7.5 7.0 6.5 6.0 5.5 5.0 4.5 4.0 3.5 3.0 2.5 2.0 1.5 1.0 0.5 ppm

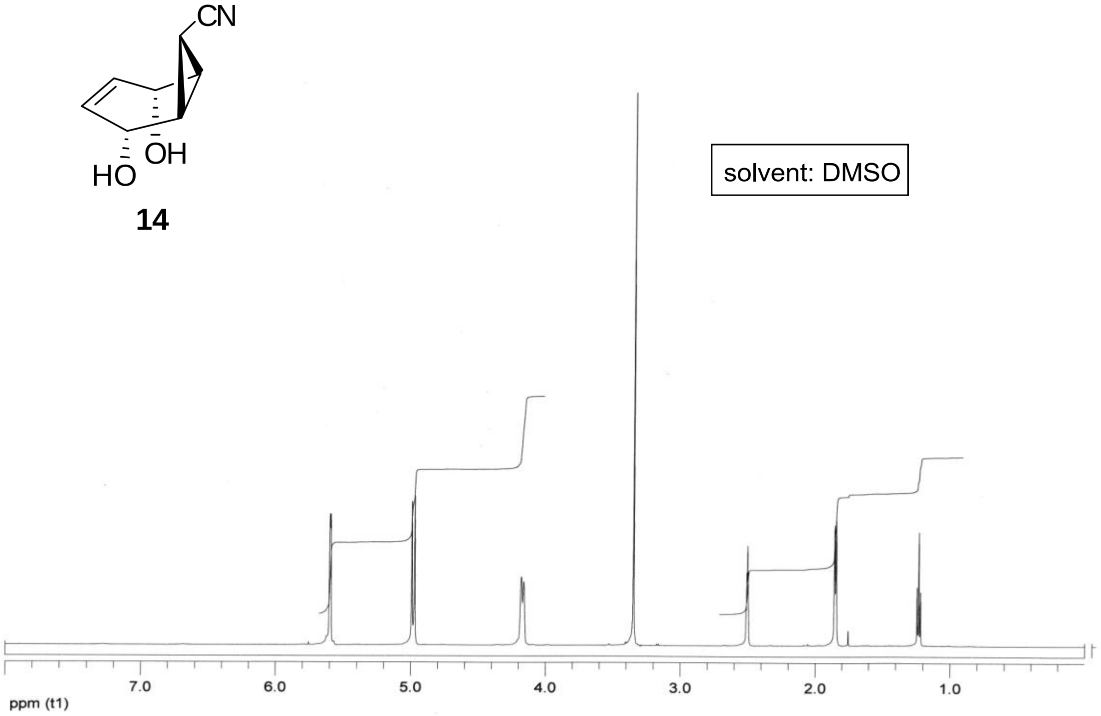
CN
OH
HO
14
solvent: DMSO
7.0
6.0
5.0
4.0
3.0
2.0
1.0
ppm (t1)

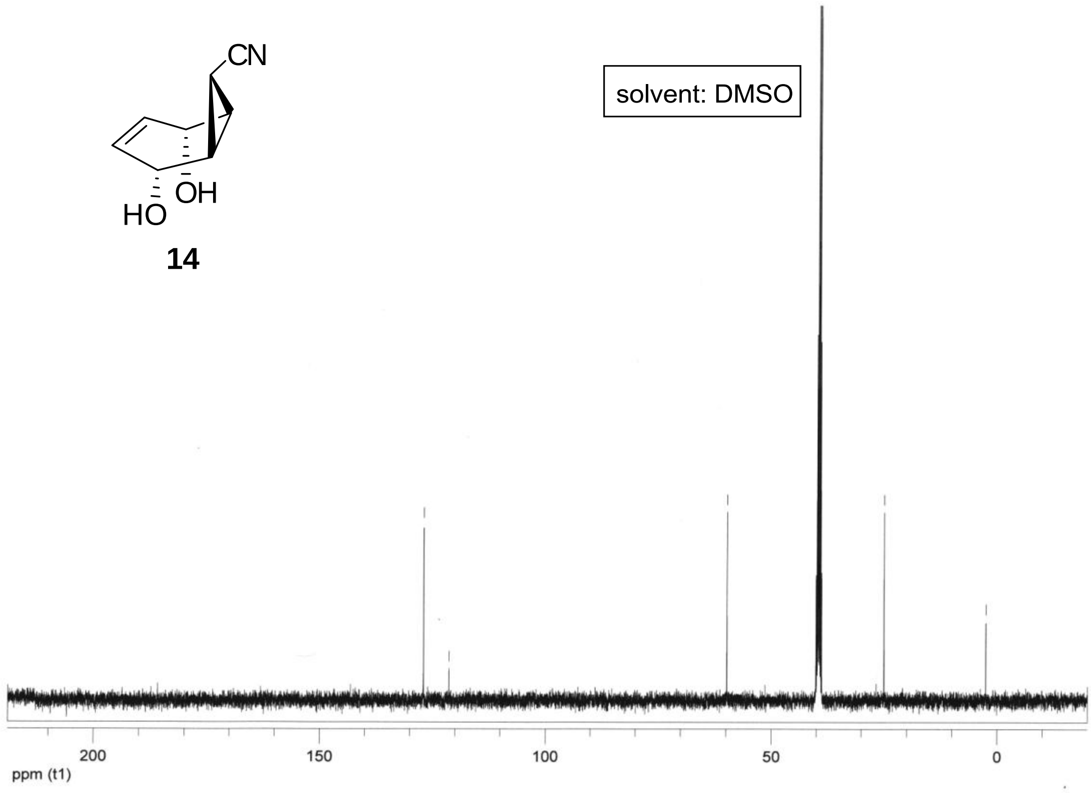
CN
OH
HO
14
solvent: DMSO
200
150
100
50
0
ppm (t1)

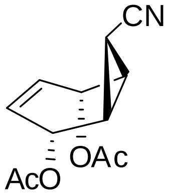
CN
OAc
AcO

15

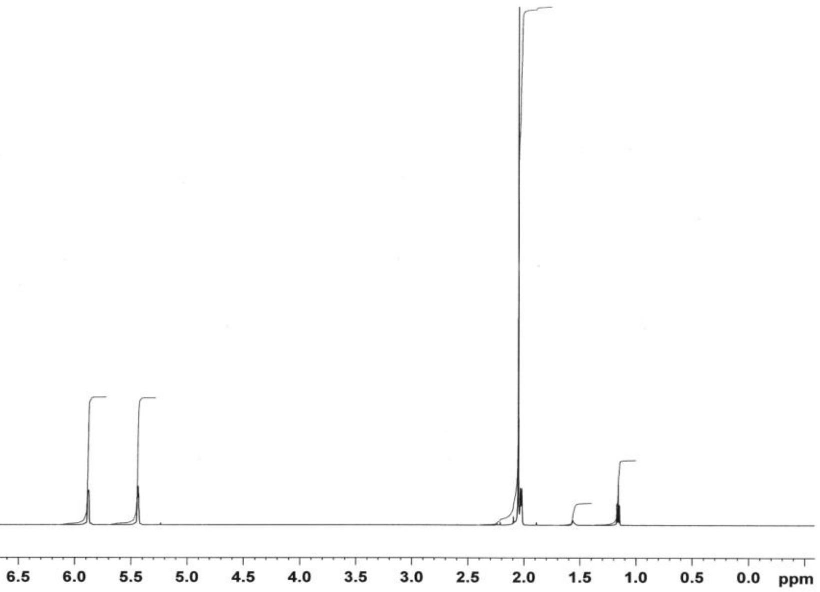
8.0 7.5 7.0 6.5 6.0 5.5 5.0 4.5 4.0 3.5 3.0 2.5 2.0 1.5 1.0 0.5 0.0 ppm

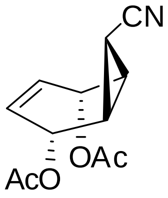
CN
OAc
AcO

15

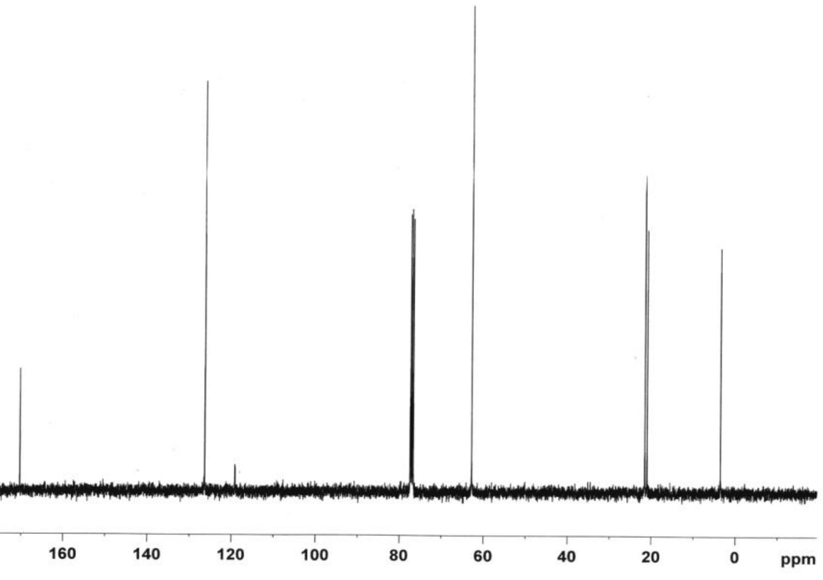
200 180 160 140 120 100 80 60 40 20 0 ppm

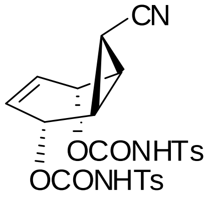
CN
OCONHTs
OCONHTs

16

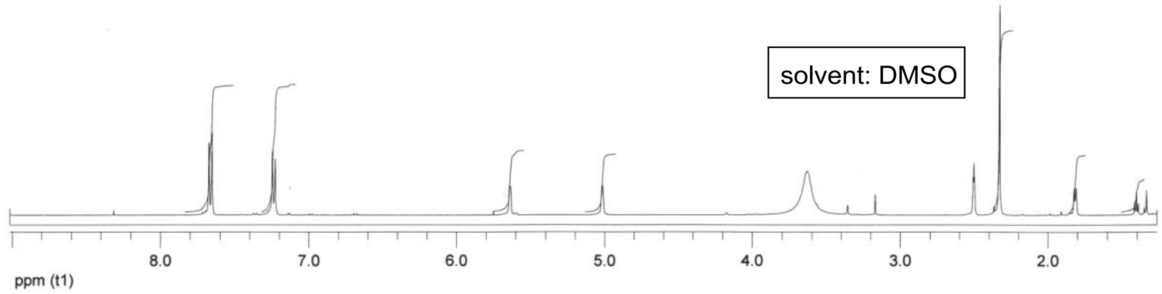
solvent: DMSO
8.0
7.0
6.0
5.0
4.0
3.0
2.0
ppm (t1)

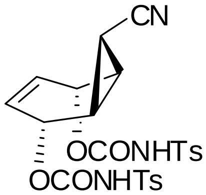
CN
OCONHTs
OCONHTs

16

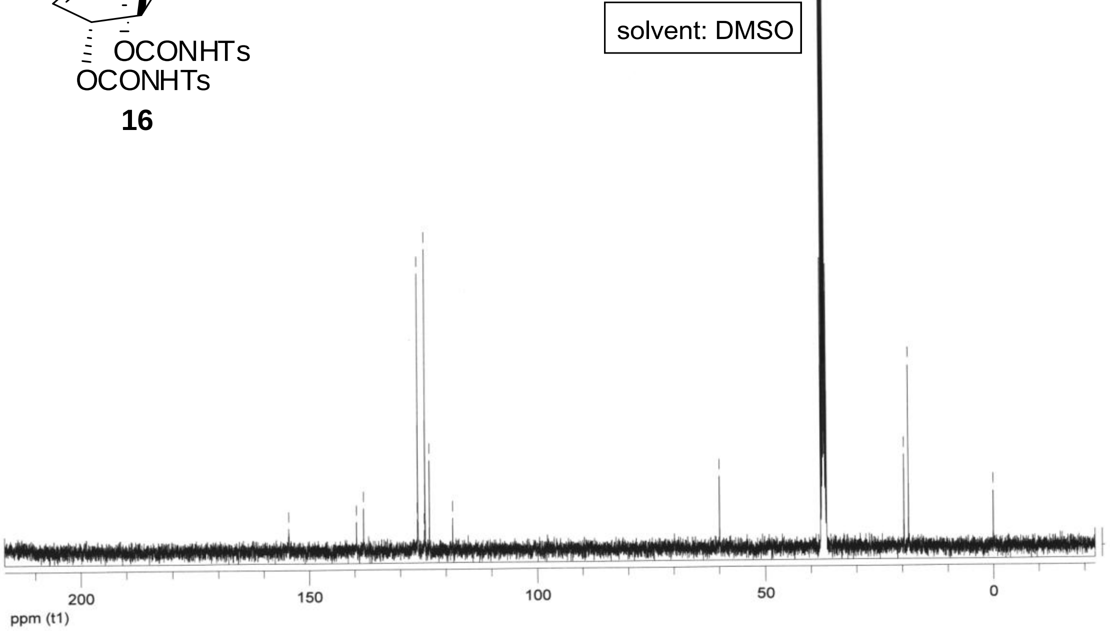
solvent: DMSO
200
150
100
50
0
ppm (t1)

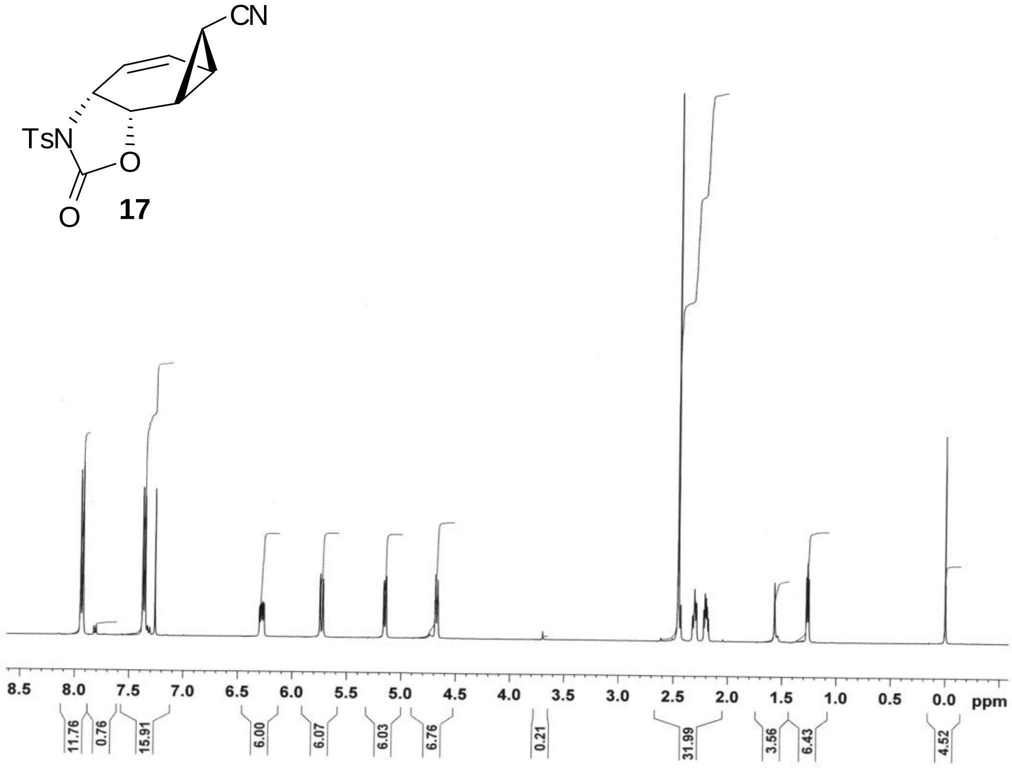
CN
TsN
O
O
17
8.5 8.0 7.5 7.0 6.5 6.0 5.5 5.0 4.5 4.0 3.5 3.0 2.5 2.0 1.5 1.0 0.5 0.0 ppm
11.76 0.76 15.91 6.00 6.07 6.03 6.76 0.21 31.99 3.56 6.43 4.52

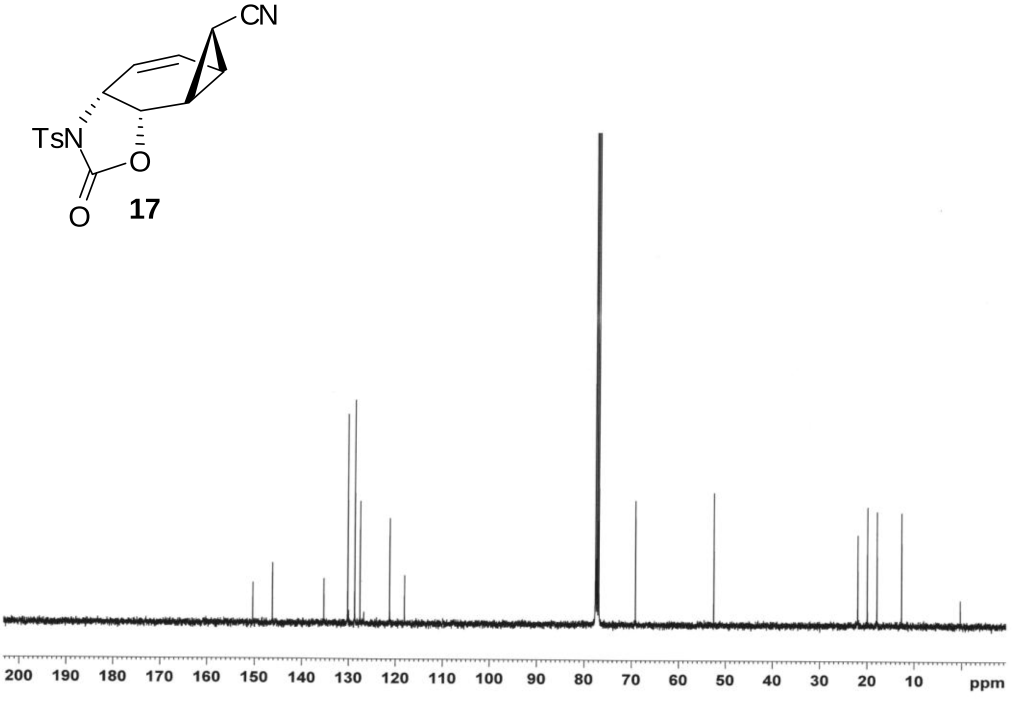
CN
TsN
O
O
17
200 190 180 170 160 150 140 130 120 110 100 90 80 70 60 50 40 30 20 10 ppm

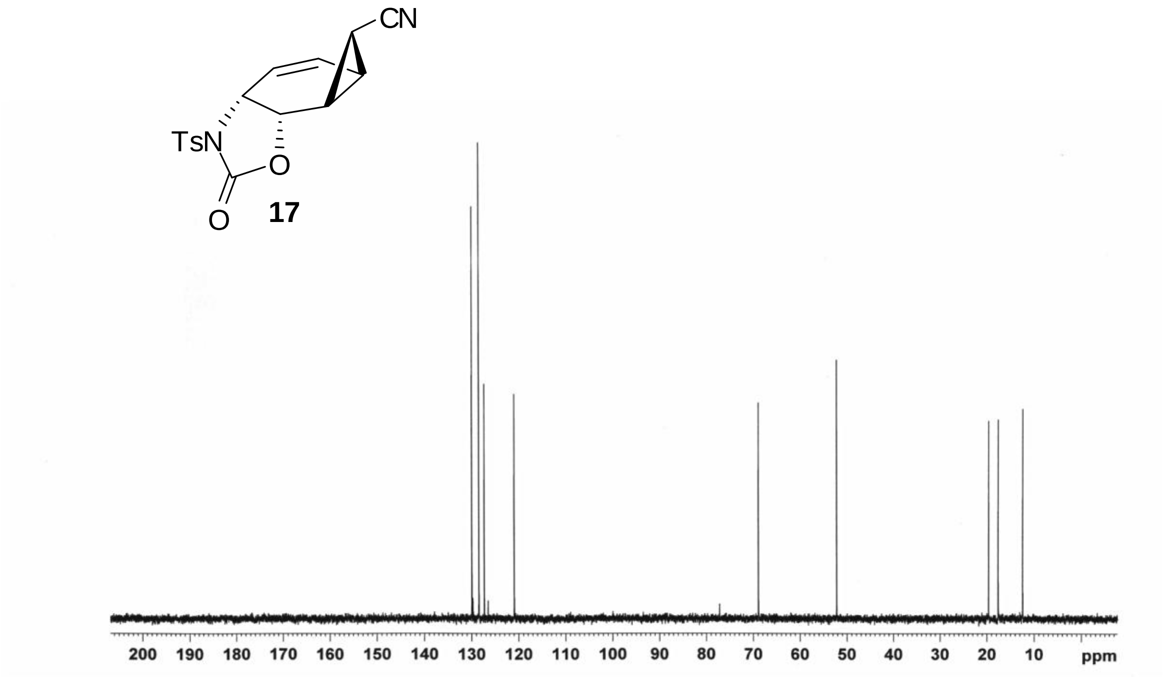
CN
TsN
O
O
200 190 180 170 160 150 140 130 120 110 100 90 80 70 60 50 40 30 20 10 ppm

17

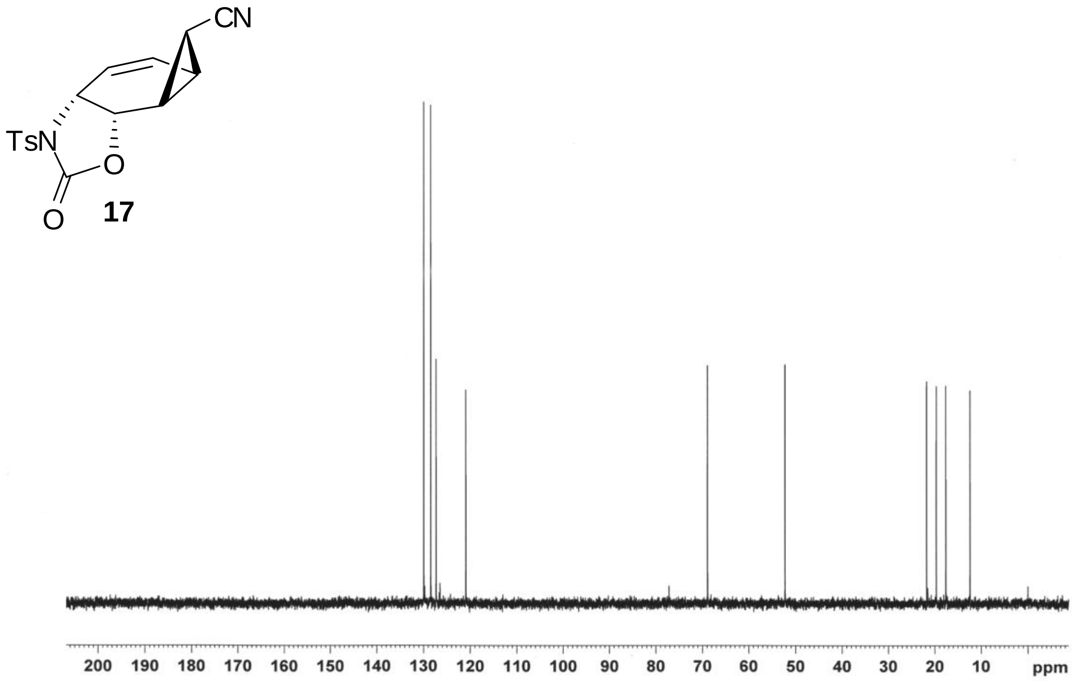
CN
TsN
O
O
200 190 180 170 160 150 140 130 120 110 100 90 80 70 60 50 40 30 20 10 ppm

17

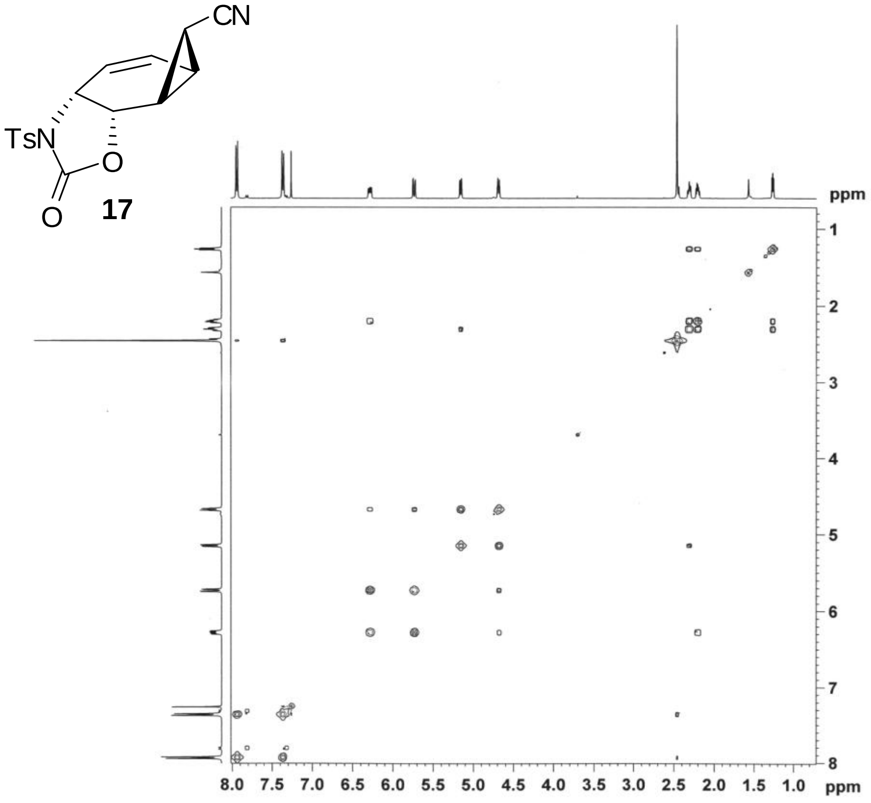
CN
TsN
O
O
ppm
1
2
3
4
5
6
7
8
8.0 7.5 7.0 6.5 6.0 5.5 5.0 4.5 4.0 3.5 3.0 2.5 2.0 1.5 1.0
ppm

17

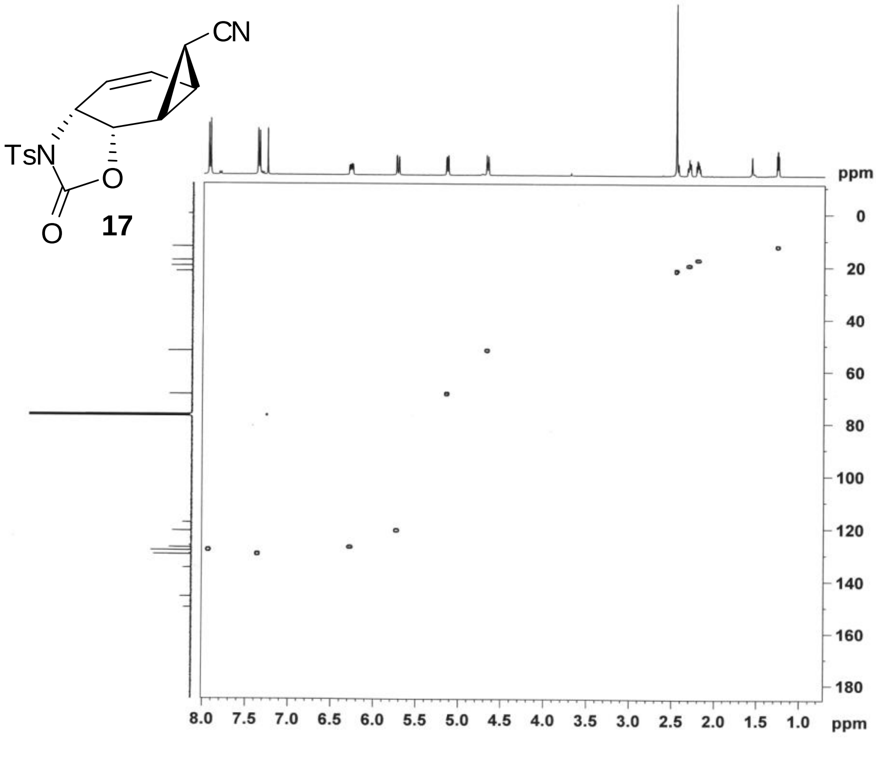
CN
TsN
O
O
ppm
0
20
40
60
80
100
120
140
160
180
8.0 7.5 7.0 6.5 6.0 5.5 5.0 4.5 4.0 3.5 3.0 2.5 2.0 1.5 1.0
ppm

17

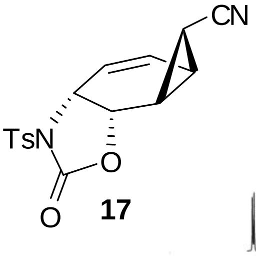
CN
TsN
O
O

17

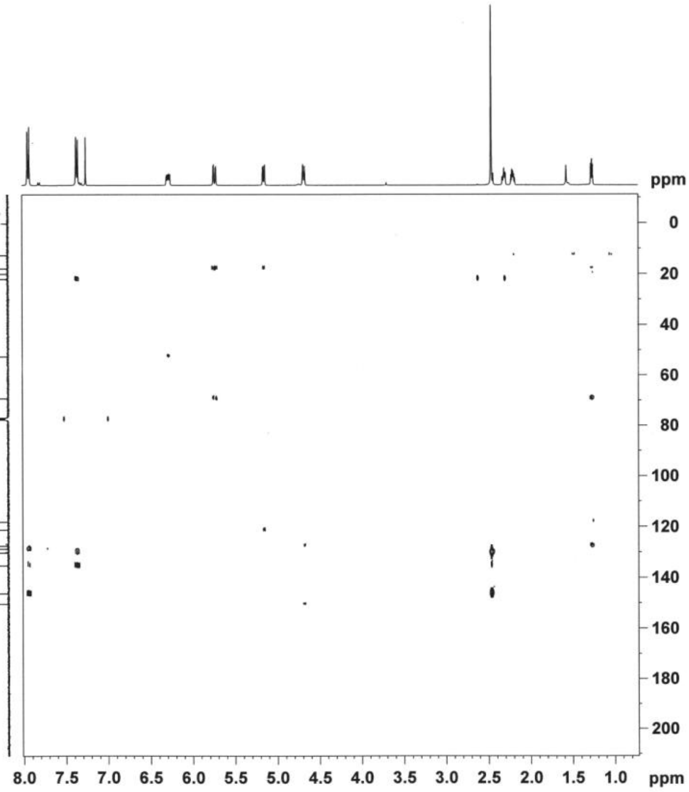
ppm
0
20
40
60
80
100
120
140
160
180
200
8.0 7.5 7.0 6.5 6.0 5.5 5.0 4.5 4.0 3.5 3.0 2.5 2.0 1.5 1.0
ppm

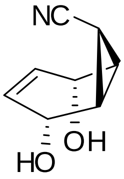
NC
OH
HO

18a

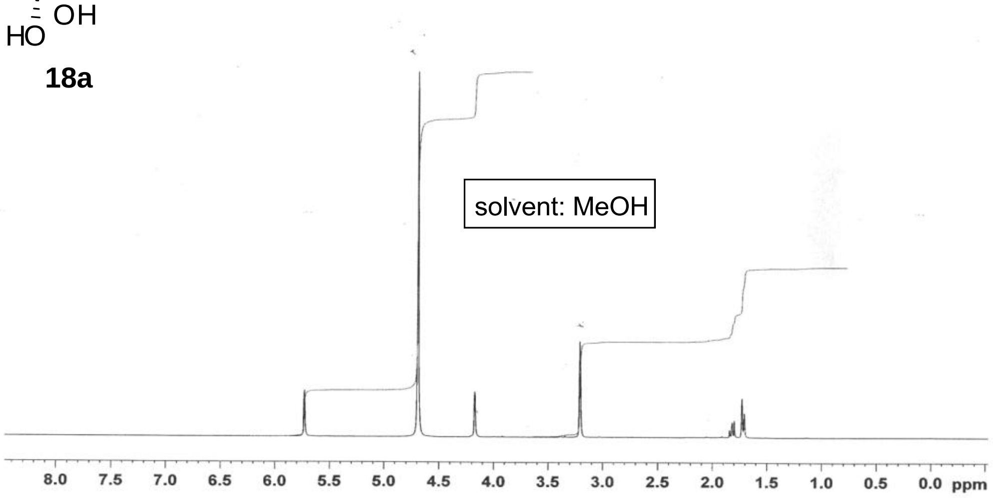
solvent: MeOH
8.0 7.5 7.0 6.5 6.0 5.5 5.0 4.5 4.0 3.5 3.0 2.5 2.0 1.5 1.0 0.5 0.0 ppm

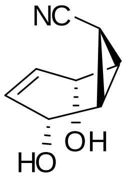
NC
OH
HO

18a

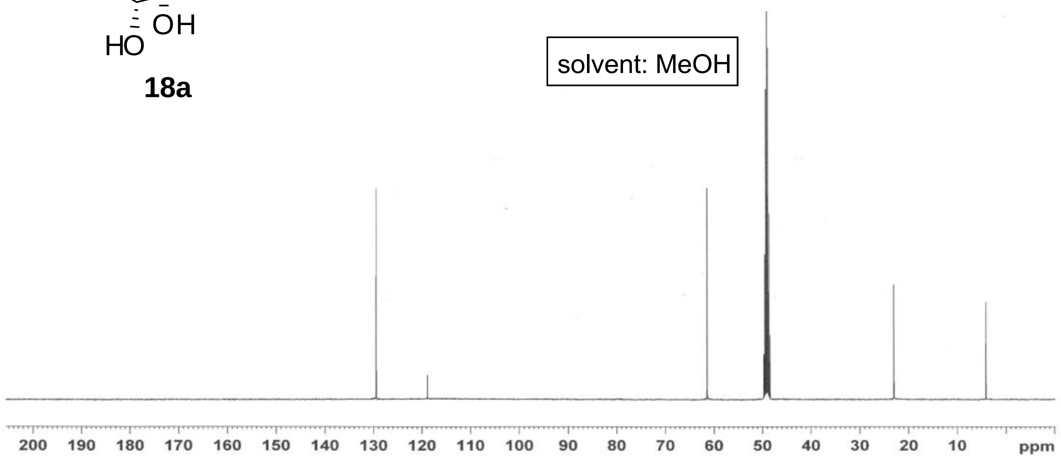
solvent: MeOH
200 190 180 170 160 150 140 130 120 110 100 90 80 70 60 50 40 30 20 10 ppm

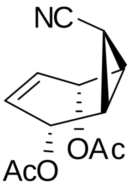

18b

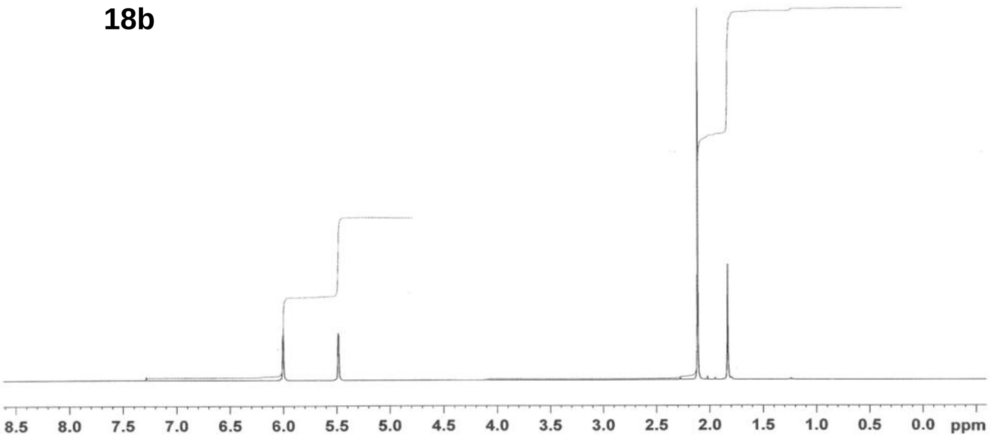

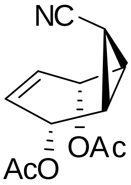

18b

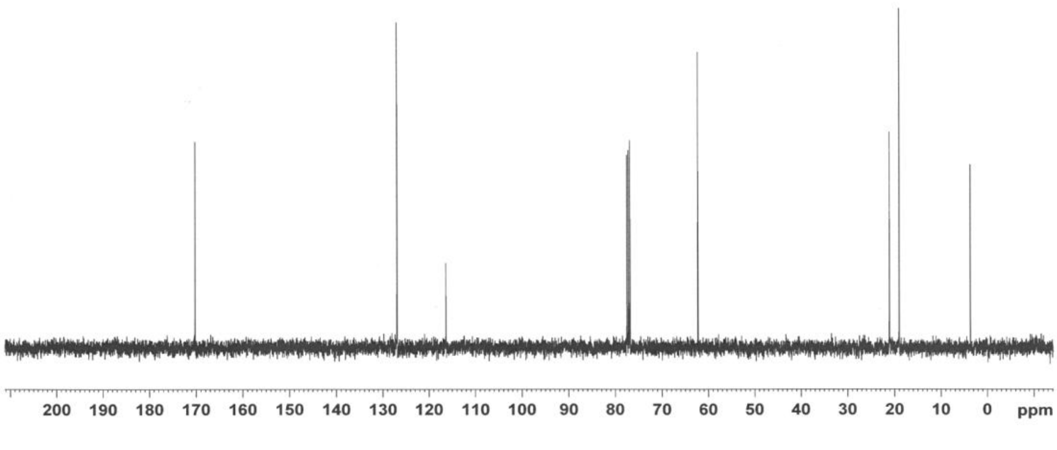

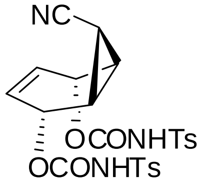
NC
OCONHTs
OCONHTs

19

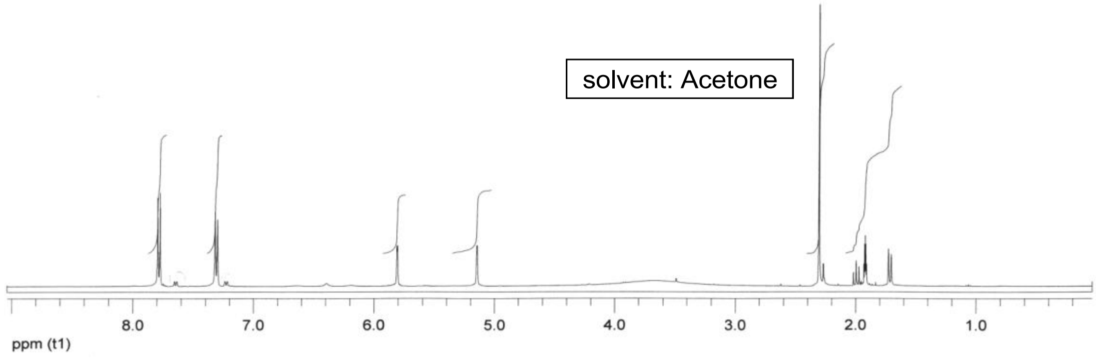
solvent: Acetone
ppm (t1)
8.0
7.0
6.0
5.0
4.0
3.0
2.0
1.0

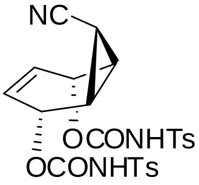
NC
OCONHTs
OCONHTs

19

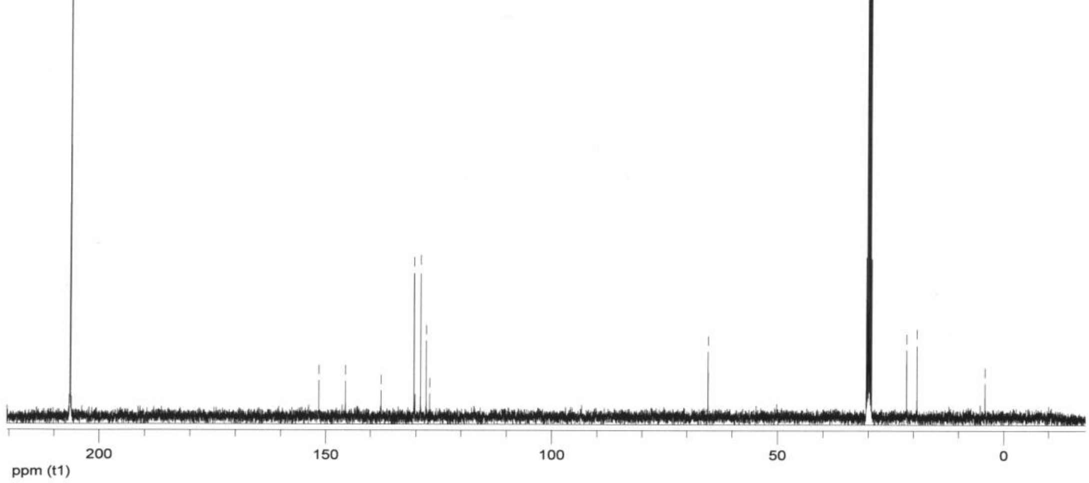
solvent: Acetone
ppm (t1)
200
150
100
50
0

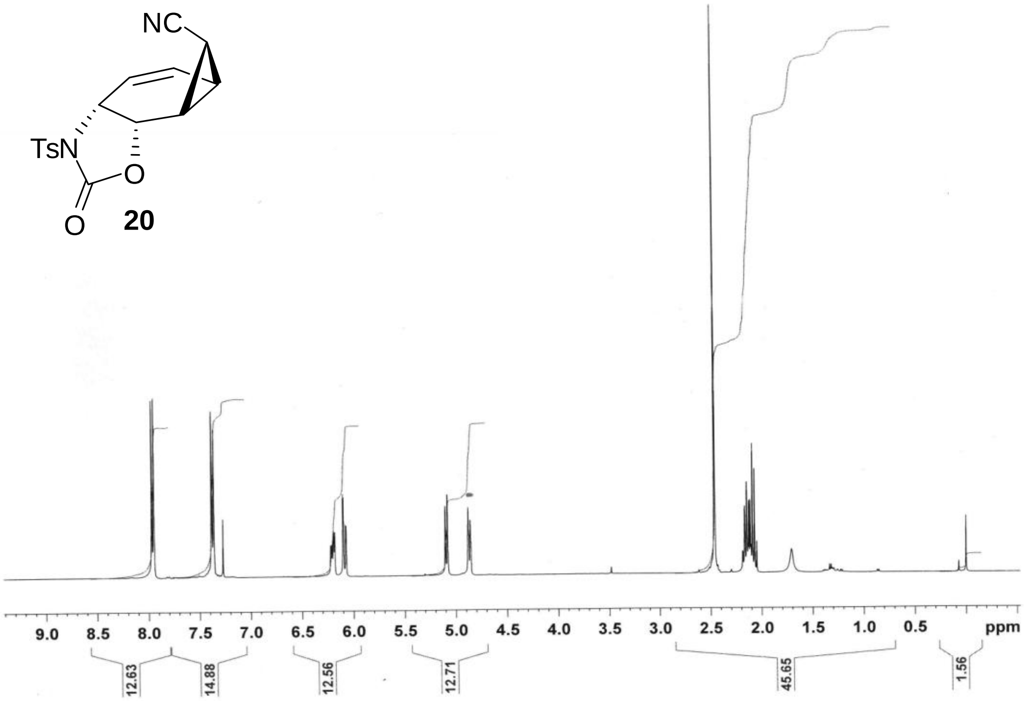
NC
TsN
O
O
20
9.0 8.5 8.0 7.5 7.0 6.5 6.0 5.5 5.0 4.5 4.0 3.5 3.0 2.5 2.0 1.5 1.0 0.5 ppm
12.63
14.88
12.56
12.71
45.65
1.56

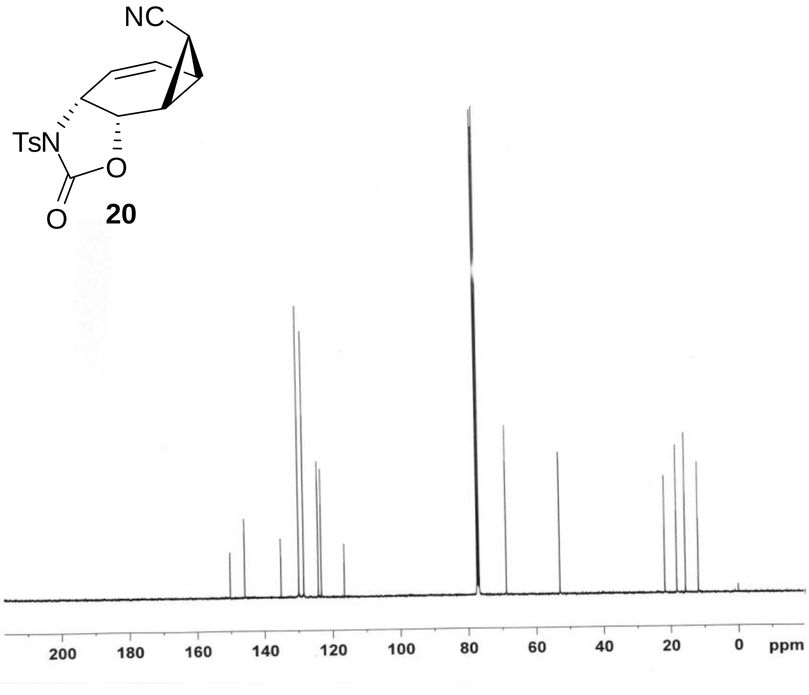
NC
TsN
O
O
20
200 180 160 140 120 100 80 60 40 20 0 ppm

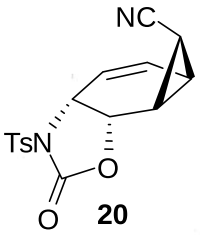

20

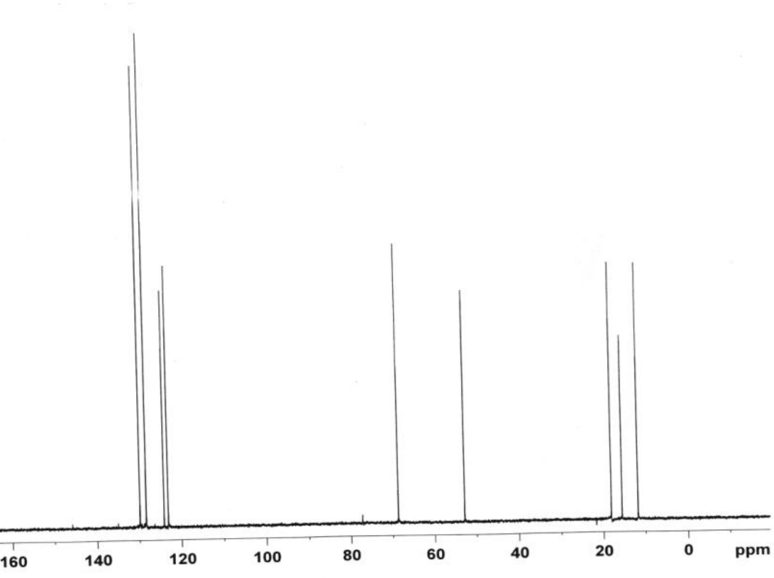

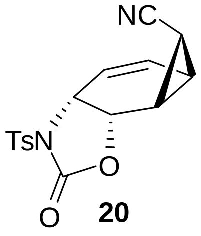
NC
TsN
O
O

20

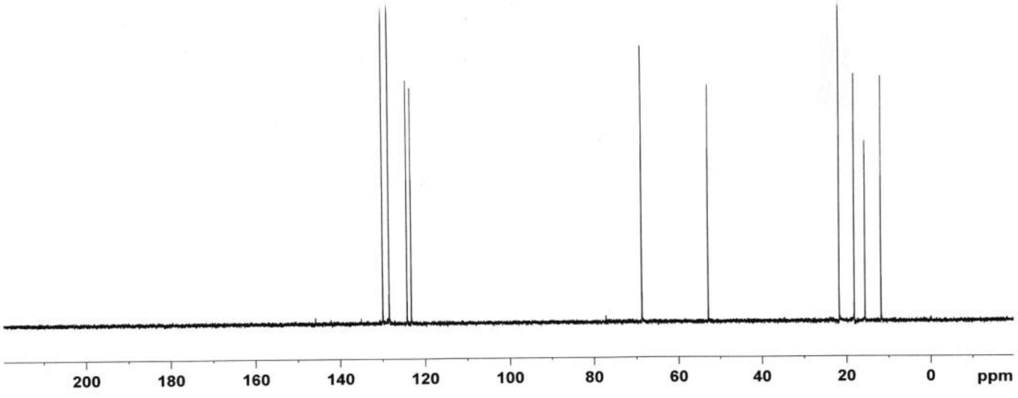
200
180
160
140
120
100
80
60
40
20
0
ppm

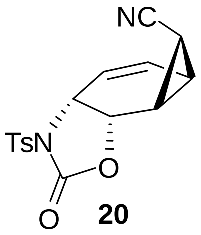
NC
TsN
O
O

20

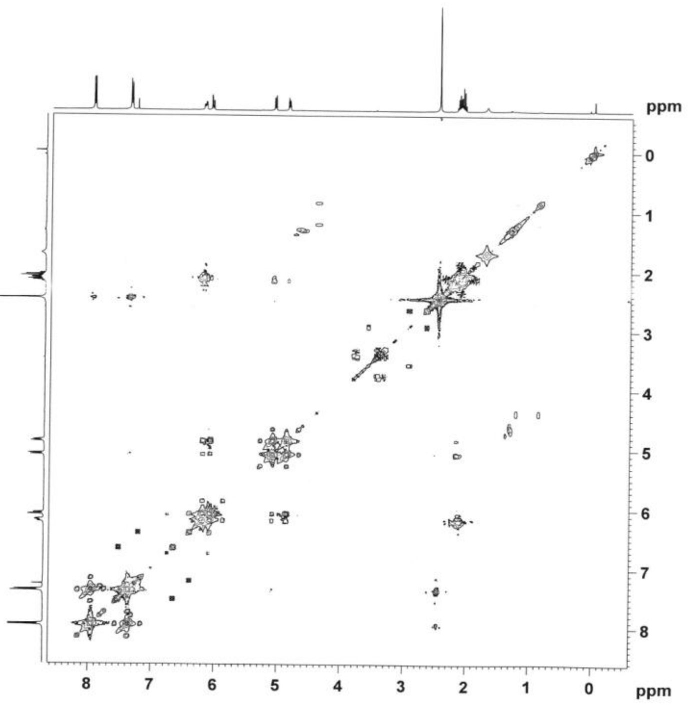
ppm
0
1
2
3
4
5
6
7
8
ppm

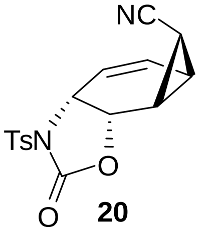
NC
TsN
O
O

20

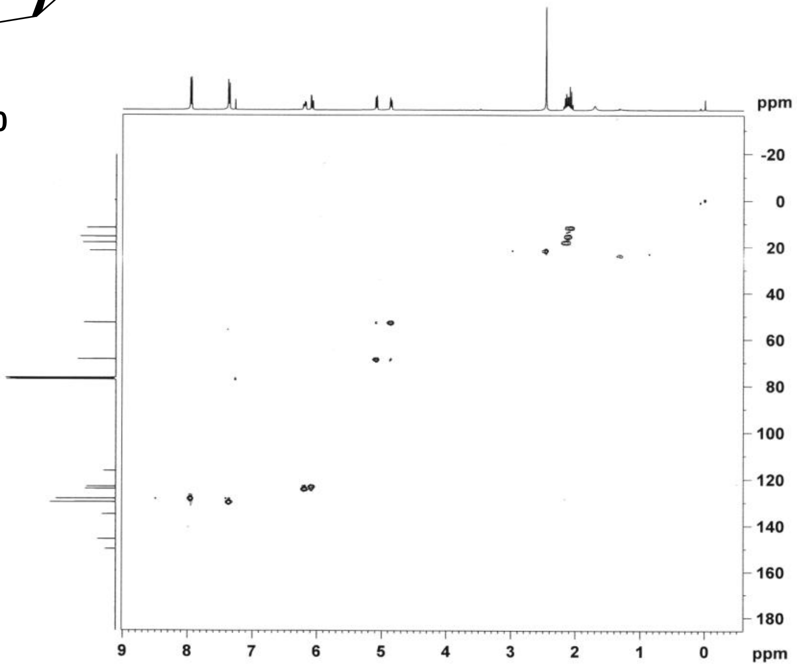
ppm
-20
0
20
40
60
80
100
120
140
160
180
9 8 7 6 5 4 3 2 1 0
ppm

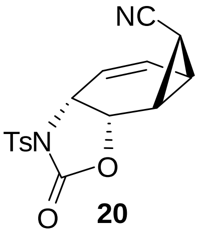
NC
TsN
O
O

20

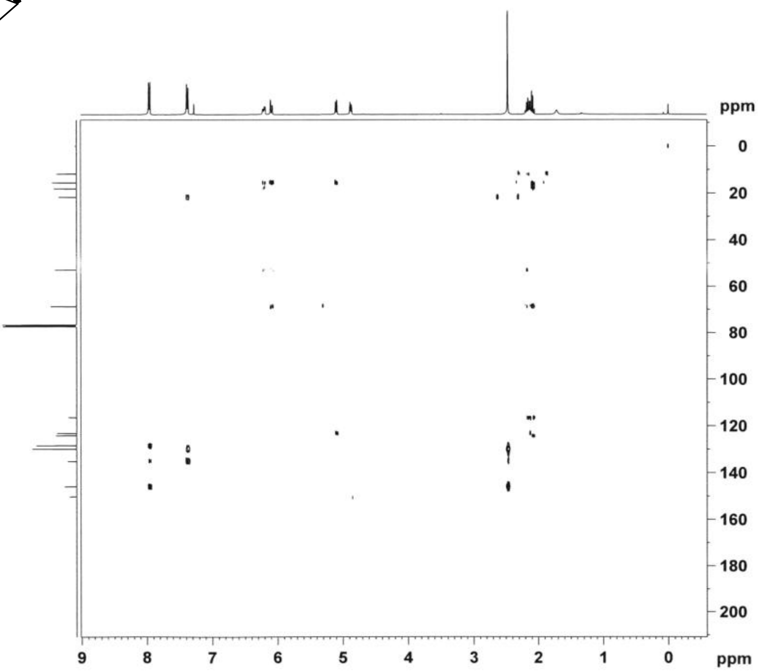
ppm
0
20
40
60
80
100
120
140
160
180
200
9 8 7 6 5 4 3 2 1 0
ppm
